# Trait-based study predicts glycerol/diol dehydratases as a key function of the gut microbiota of hindgut-fermenting carnivores

**DOI:** 10.1186/s40168-024-01863-4

**Published:** 2024-09-19

**Authors:** Qing Li, Hans-Joachim Ruscheweyh, Lærke Hartmann Østergaard, Micael Libertella, Kim Skalborg Simonsen, Shinichi Sunagawa, Alberto Scoma, Clarissa Schwab

**Affiliations:** 1 Department of Biological and Chemical Engineering, Aarhus University, Gustav Wieds Vej 10, 8000 Arhus, Denmark; 2 Department of Biology, Institute of Microbiology and Swiss Institute of Bioinformatics, ETH Zürich, Vladimir-Prelog-Weg 4, 8093 Zurich, Switzerland; 3 Givskud Zoo - Zootopia, Løveparkvej 3, 7323 Givskud, Denmark; 4 Present address: National Food Institute, Technical University of Denmark, Kgs. Lyngby, Denmark

**Keywords:** Diet, Gut physiology, Glycerol diol dehydratase, Metagenomic sequencing, Biomarker

## Abstract

**Background:**

Microbial *pdu* and *cob-cbi-hem* gene clusters encode the key enzyme glycerol/diol dehydratase (PduCDE), which mediates the transformation of dietary nutrients glycerol and 1,2-propanediol (1,2-PD) to a variety of metabolites, and enzymes for cobalamin synthesis, a co-factor and shared good of microbial communities. It was the aim of this study to relate *pdu* as a multipurpose functional trait to environmental conditions and microbial community composition. We collected fecal samples from wild animal species living in captivity with different gut physiology and diet (*n* = 55, in total 104 samples), determined occurrence and diversity of *pdu* and *cob-cbi-hem* using a novel approach combining metagenomics with quantification of metabolic and genetic biomarkers, and conducted in vitro fermentations to test for trait-based activity.

**Results:**

Fecal levels of the glycerol transformation product 1,3-propanediol (1,3-PD) were higher in hindgut than foregut fermenters. Gene-based analyses indicated that *pduC* harboring taxa are common feature of captive wild animal fecal microbiota that occur more frequently and at higher abundance in hindgut fermenters. Phylogenetic analysis of genomes reconstructed from metagenomic sequences identified captive wild animal fecal microbiota as taxonomically rich with a total of 4150 species and > 1800 novel species but pointed at only 56 species that at least partially harbored *pdu* and *cbi-cob-hem*. While taxonomic diversity was highest in fecal samples of foregut-fermenting herbivores, higher *pduC* abundance and higher diversity of *pdu/cbi-cob-hem* related to higher potential for glycerol and 1,2-PD utilization of the less diverse microbiota of hindgut-fermenting carnivores in vitro.

**Conclusion:**

Our approach combining metabolite and gene biomarker analysis with metagenomics and phenotypic characterization identified Pdu as a common function of fecal microbiota of captive wild animals shared by few taxa and stratified the potential of fecal microbiota for glycerol/1,2-PD utilization and cobalamin synthesis depending on diet and physiology of the host. This trait-based study suggests that the ability to utilize glycerol/1,2-PD is a key function of hindgut-fermenting carnivores, which does not relate to overall community diversity but links to the potential for cobalamin formation.

Video Abstract

**Supplementary Information:**

The online version contains supplementary material available at 10.1186/s40168-024-01863-4.

## Introduction

While ancestral mammals were carnivores, the transition from carnivores to herbivores was a significant milestone in mammalian history [1]. This evolutionary shift facilitated the proliferation of herbivorous species, which now constitute approximately 80% of mammals. Adaption to a herbivorous diet related to a longer gut retention time through an enlargement of the foregut or hindgut [1] enabling the intestinal microbial communities to access, degrade, and ferment plant-based feedstock. Today, most omnivores and carnivores are hindgut fermenters while herbivores ferment in the foregut or hindgut [2]. Fecal microbial communities differ between foregut- and hindgut-fermenting herbivores, and hindgut-fermenting omnivores and carnivores [2], and diet explained the α- and β-diversity variation to a larger extent than geography, habitat, genome-based host phylogeny, and technical factors [3]. Concurrently, microbial genes and metabolites recovered from mammalian feces were significantly clustered by diet and gut physiology [3, 4] highlighting that environmental host parameters link to intestinal microbial activity and functionality. Fecal microbiota is frequently used as a proxy of intestinal microbial communities [2, 3, 5].

**Fig. 1:**
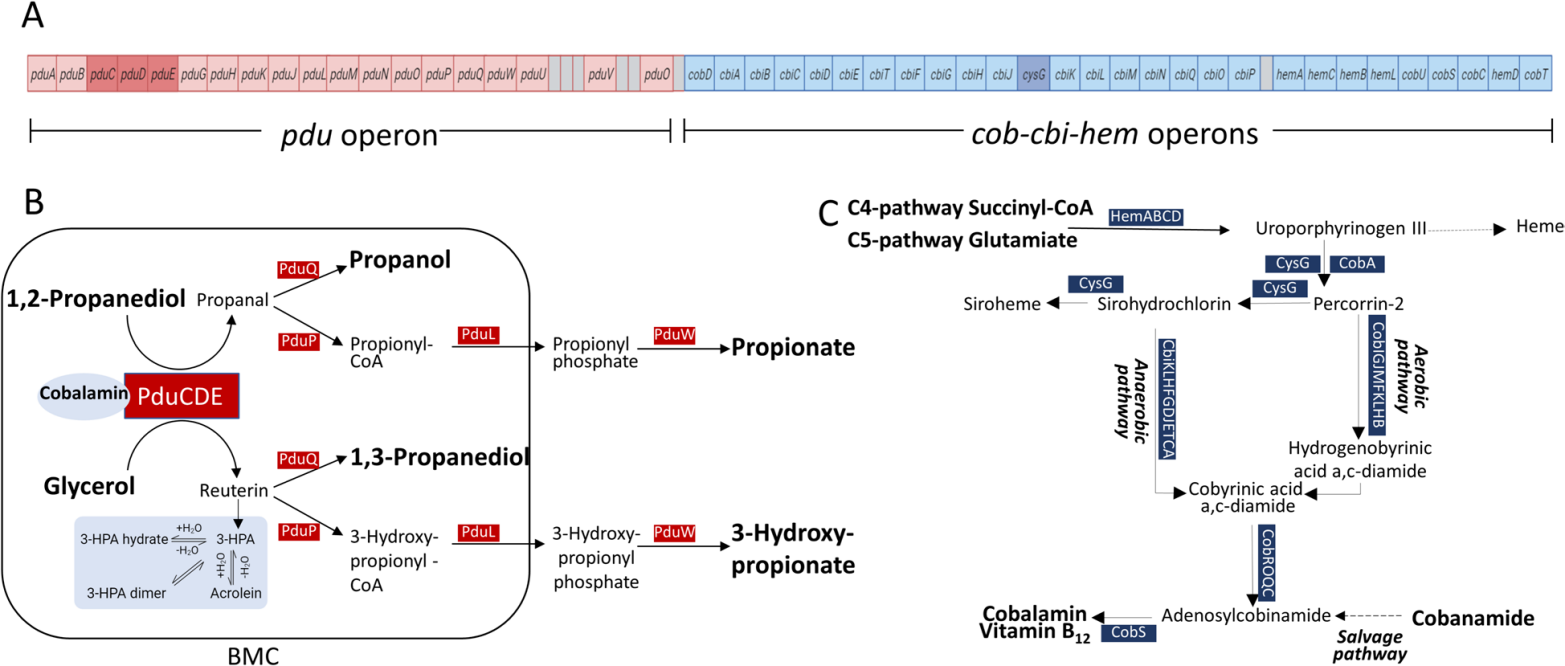
Pdu and cobalamin biosynthesis pathways. **A** Gene structure of *pdu/cob-cbi-hem* operons in *L. reuteri* with highlighted key genes [8]. Genes were not drawn according to scale. **B** Pdu encoded by the *pdu* operon mediates the degradation of 1,2-propanediol and glycerol to form propanol and propionate, and 1,3-propanediol and 3-hydroxypropionate as final metabolites, respectively. **C** Cobalamin synthesis pathways are encoded by genes on *cob-cbi-hem* operons

The operons *pdu* and *cob-cbi-hem* (Fig. 1A) were previously identified as host-related traits of the gut symbiont *Limosilactobacillus reuteri* indicating a possible link between microbial function and intestinal microbial lifestyle [6]. The key enzyme of *pdu*, glycerol/propanediol dehydratase (PduCDE) encoded by *pduCDE* genes, catalyzes the transformation of 1,2-propanediol (1,2-PD) to propanal, which can be further metabolized to propanol and to the short-chain fatty acid (SCFA) propionate mediated by enzymes of the Pdu pathway (Pdu, Fig. 1B). Propionate provides energy to the host, modulates the immune system, and promotes intestinal homeostasis [7]. As a second substrate, PduCDE uses glycerol to form 3-hydroxypropanal with the end products 1,3-propanediol (1,3-PD) and 3-hydroxypropionate [8]. 3-Hydroxypropanal can spontaneously degrade to the double unsaturated reactive aldehyde acrolein; both compounds are part of the antimicrobial reuterin system [9]. Part of the Pdu metabolism occurs in bacterial proteinaceous microcompartments (BMC) formed by PduA, PduB, and PduJ [10]. Such BMC enhance enzyme efficiency and protect bacteria from toxic intermediates like propanal [11]. Both substrates of PduCDE are readily available in gut ecosystems as degradation products of triglycerides (glycerol) and as an intermediate of the microbial metabolism of the deoxyhexoses fucose and rhamnose (1,2-PD) [12]. Fucose and rhamnose are components of dietary plant and host-derived glycans, e.g., pectin and mucus [13, 14].

Cobalamin, a cobamamide, is a cofactor for PduCDE and other enzymes involved in the metabolism of nucleic acids, amino acids, and fatty acids [15]. Microbes can synthesize cobalamin via anaerobic, aerobic, C4/C5, and salvage pathways (Fig. 1C). In *L. reuteri* and other bacterial species, cobalamin forming enzymes are encoded by *cob-cbi-hem*, which is adjacent to the *pdu* operon [16, 17] (Fig. 1A). It has been predicted that > 80% of the members of gut and environmental microbial communities depend on external supply of cobamides, while only 30–40% have the capacity for synthesis [15].

**Fig. 2:**
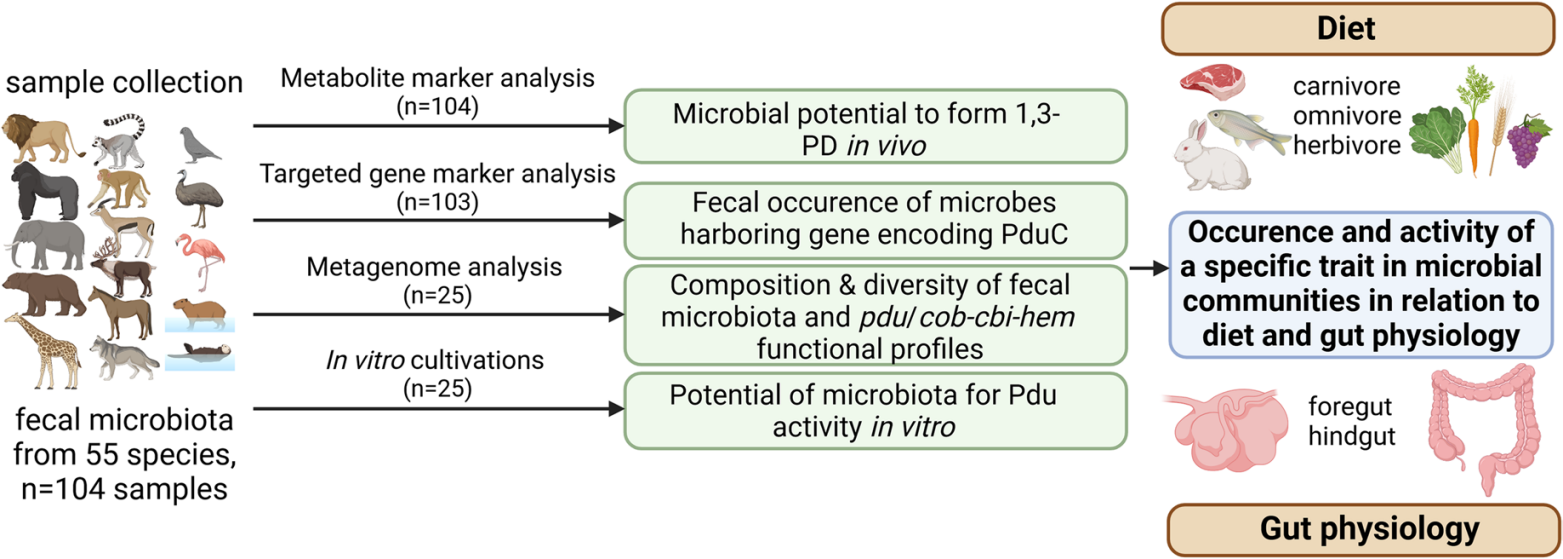
Study design. This study utilized fecal microbiota collected from 55 animal species housed at Givskud and Copenhagen Zoo (Denmark) to conduct metabolic and genetic marker analysis, metagenomic sequencing, and in vitro cultivations. This figure was created using BioRender

Due to its intrinsic relationship with gut symbiont lifestyle, dietary nutrients, and microbial interactions, *pdu* and *cob-cbi-hem* operons can be considered as highly relevant functions to study the distribution and role of specific traits of intestinal microbial communities. Using a novel multi-pronged approach, the aim of this study was to elucidate the occurrence and function of *pdu* and *cob-cbi-hem* operons of different gut microbial ecosystems. We used an experimental set-up (Fig. 2) based on *n* = 104 fecal microbial communities collected from 55 species of captive *Mammalia* and *Aves* covering different diet schemes (herbivorous, carnivorous, omnivorous) and gut physiology (foregut and hindgut fermenters) in combination with quantitative PCR and metabolite analysis to identify fecal biomarkers (Table S1), with metagenomics to determine taxonomic composition and to predict functional potential encoded by *pdu* and *cbi-cob-hem*, and with strict anaerobic fermentation to determine Pdu activity in vitro (Fig. 2).

## Results and discussion

### Occurrence of the glycerol transformation metabolite 1,3-PD links to diet and to fecal microbial communities of hindgut fermenters

Pdu driven metabolism leads to the formation of the SCFA propionate and of the glycerol-specific metabolite 1,3-PD. To identify the potential of captive wild animal gut microbiota for Pdu activity, fecal metabolite profiles of 104 fecal samples were assessed for 1,3-PD and primary SCFAs (e.g., acetate, propionate, butyrate) using HPLC-RI (Table 1, Table S1).

**Table 1:** SCFA and 1,3-propanediol levels and occurrence in feces. Fecal levels of major SCFAs (acetate, propionate, and butyrate) and 1,3-propanediol (1,3-PD) were determined with HPLC-RI

Factor		Fecal SCFA and 1,3-PD (median; 25 and 75% quartile)							
		**Acetate**		**Propionate**		**Butyrate**		**Total SCFA**	**1,3-PD**
		Levels (µmol g^−1^)	Proportion (%)	Levels (µmol g^−1^)	Proportion (%)	Levels (µmol g^−1^)	Proportion (%)	Levels (µmol g^−1^)	Levels (µmol g^−1^)
**Gut physiology**	Foregut	25.4; 16.8; 34.4	85.2; 82.6; 87.1	0; 0; 0.7	0; 0; 1.9	3.9; 3.4; 4.8	13.1; 10.6; 15.1	30.0; 20.4; 39.2	0; 0; 5.0
	Hindgut	37.4^**^; 19.8; 61.0	84.7; 77.4, 98.1	0; 0; 2.6	0; 0; 6.0	4.5; 0; 6.4	11.2; 0; 16.1	46.7^**^; 23.4; 73.7	4.6^**^; 0; 6.3
**Diet**	Carnivores	31.3^ab^; 12.3; 62.0	86.2^ab^; 67.5; 100	0; 0; 4.7	0; 0; 5.3	4.3; 0; 7.5	12.1^ab^; 0; 18.0	33.6^ab^; 17.2; 73.9	4.8^ab^; 0; 6.2
	Omnivores	55.7^a^; 30.3; 95.4	89.2^a^; 82.0; 100.0	0; 0; 1.3	0; 0; 4.3	3.7; 0; 9.6	6.1^bc^; 0; 11.8	71.9^a^; 32.6; 104.2	5.3^a^; 0; 7.4
	Herbivores	26.0^bc^; 18.2; 37.0	84.4^bc^; 79.9; 87.0	0; 0; 1.7	0; 0; 4.1	4.1; 3.5; 4.2	13.6^a^; 10.0; 16.3	31.0^bc^; 23.0; 44.7	0; 0^bc^; 5.4

* indicates significant differences in abundance and occurrence of (*p* < 0.01) between hindgut-fermenting animals and foregut-fermenting animals. Amount and percentage of SCFAs and 1,3-PD from feces of carnivores, omnivores, and herbivores do not share a common lowercase superscript differ significantly (*p* < 0.05)

Total SCFA contents ranged from 4.9 to 188.8 µmol∙g^−1^, with acetate contributing the highest proportion (range 50.9 to 100%), followed by butyrate (0 to 40.4%) and propionate (0 to 31.5%) (Table 1). The concentration of acetate was higher in fecal samples of hindgut than foregut fermenters (37.4 vs. 25.4 µmol∙g^−1^, *p* < 0.01), while concentration and proportion of acetate was higher in samples of omnivores rather than herbivores (55.7 vs. 26.0 µmol∙g^−1^, *p* < 0.001, 89.3 vs. 84.0%, *p* < 0.05). The proportion of butyrate was higher in herbivores than in omnivores (13.7 vs. 6.1%, *p* < 0.001) (Table 1), and it was suggested before that diet is a major determinant in defining composition of the fecal butyrate producing community of animals [18].

In foregut fermenters, microbial degradation and fermentation almost exclusively occur in the upper gastrointestinal tract, while in hindgut fermenters microbes are most abundant and active in the large intestine, with overall shorter retention times [19]. This systematic difference might at least partly explain the higher acetate levels determined in fecal samples of hindgut- than of foregut-fermenting animals. Concentrations or proportions of propionate were not affected by diet or physiology, possibly because propionate cannot only be formed from 1,2-PD but also from lactate and succinate [20].

The glycerol metabolite 1,3-PD was detected in 56% of the samples at levels ranging from 4.5 to 14.6 µmol∙g^−1^ (Table 1). 1,3-PD occurred more frequently (68.0 vs. 33.3%, *p* < 0.05) and at higher median levels (5.3 vs. 0.0 µmol∙g^−1^, *p* < 0.01) in fecal samples of omnivores than herbivores. 1,3-PD was detected in 60.0% of the samples collected from carnivores (median level 4.8 µmol∙g^−1^). The fecal concentration of 1,3-PD was higher in hindgut than foregut fermenters (median level 4.6 vs. 0.0 µmol∙g^−1^, *p* < 0.01). Glycerol is the only (known) carbon source that acts as substrate for the microbial biosynthesis of 1,3-PD. A higher content of triglycerides in the meat-containing diet of omnivores and carnivores as compared to herbivores, together with differences in lipid digestion efficiency [21], can lead to higher glycerol levels in the hindgut and thus to the microbial formation of 1,3-PD. Considering 1,3-PD as a diet- and substrate-dependent marker of *pdu* metabolism, hindgut-fermenting carnivores and omnivores showed higher glycerol transformation than foregut-fermenting herbivores.

### Occurrence and abundance of *pduC* contributing taxa was higher in hindgut than foregut fermenters

In parallel to fecal metabolite analysis, qPCR was performed on fecal microbial communities targeting *pduC* as a genetic marker of PduCDE function (Table S2). We quantified taxa known for the presence of *pdu* that were abundant in fecal microbiota of humans, which can be considered a hindgut-fermenting omnivore. The tested taxa included *L. reuteri*, *Anaerobutyricum hallii*, *Blautia obeum*, *Veillonella dispar*, *Flavonifractor plautii*, and *Ruminococcus gnavus* [22, 23]. We also included *Clostridium perfringens*, which was identified in carnivores/wild captive animals before and used glycerol via PduCDE [18, 24, 25] (Suppl. Methods).

The majority of samples (85.4%) harbored at least one taxon carrying *pduC* (median 2) with an abundance ≥ 4.7 log *pduC* g^−1^ (Table S1), indicating the common occurrence of *pduC* harboring taxa in fecal microbiota of captive wild animals. The total abundance of tested *pduC* taxa was higher in hindgut fermenting or carnivore microbiota than foregut fermenting (median level 5.5 vs. 4.9 log *pduC*∙g^−1^, *p* < 0.001) or omnivore and herbivore microbiota (median level 8.5 vs. 5.2 vs. 5.2 log *pduC*∙g^−1^, *p* < 0.05) (Table 2). The significantly (*p* < 0.001) most recurrent *pduC*-harboring taxon was *L. reuteri* (66.3% of samples) (Table 2, Table S1), in agreement with recent studies linking the presence of *pdu/cob-cbi-hem* to omnivorous (human, primates, pigs, and chicken) and herbivorous hosts [6, 26]. Yet, *L. reuteri* generally had low abundance (median level 5.4 log *pduC* g^−1^).

**Table 2:** Overview of *pduC* abundance and occurrence from selected bacteria of fecal microbiota by qPCR. The cell copies major taxa known to harbour *pduC*

Taxon	Factor	Cell counts (median; 25 and 75% quartile; log *pduC*∙g^−1^)/occurrence (%)													
		*Anaerobutyricum hallii*		*Limosilactobacillus reuteri*		*Ruminococcus gnavus*		*Veillonella dispar*		*Blautia obeum*		*Clostridium perfringens*		Sum	
		Median	Occurrence	Median	Occurrence	Median	Occurrence	Median	Occurrence	Median	Occurrence	Median	Occurrence	Median	Occurrence
**Gut physiology**	Foregut	0; 0, 0	19.0	4.8; 0, 4.9	54.8	0^**^; 0, 0	11.9^*^	0; 0, 0	14.3	0; 0, 0	4.8	0; 0, 0	0	4.9; 4.7, 5.2	76.2
	Hindgut	0^**^; 0, 6.5	45.9^*^	4.8; 0; 4.9	75.4	0; 0, 0	0	0; 0, 0	23.0	4.8^***^; 0, 4.9	50.8^***^	0^**^; 0, 0	14.8^*^	5.5^***^; 5.1, 7.8	90.3
**Diet**	Carnivores	0; 0, 0	20.0	4.8; 4.7, 4.9	80.0	0; 0, 0	0	0; 0, 0	20.0	4.9^a^; 1.2, 4.9	70.0^a^	8.5^a^; 0, 9.1	60.0^a^	8.5^a^; 5.9, 9.1	90.0
	Omnivores	0; 0, 5.0	33.3	4.8; 0, 4.9	70.8	0; 0, 0	0	0; 0, 4.9	29.2	0^b^; 0, 4.9	41.2^ab^	0^b^; 0, 0	4.2^b^	5.2^b^; 4.9, 6.5	88.0
	Herbivores	0; 0, 5.0	37.7	4.8; 0, 4.9	63.8	0; 0, 0	7.2	0; 0, 0	15.9	0^b^; 0, 0	23.2^b^	0^b^; 0, 0	2.9^b^	5.2^b^; 4.8, 6.2	82.6
**Total occurrence**		34.6%		63.3%		4.8%		19.2%		31.7%		8.7%		85.4%	

*, **, and *** represent higher abundance and occurrence of *p* < 0.05, *p* < 0.01, and *p* < 0.001, respectively, between hindgut-fermenting animals and foregut-fermenting animals. Abundance and occurrence of *pduC* from selected bacteria identified in fecal microbiota of carnivores, omnivores, and herbivores do not share a common lowercase superscript differ significantly (*p* < 0.05). Analysis was based on qPCR results from 103 fecal samples without one of Barbary macaque (sample nr. 9)

The omnipresent *A. hallii* species contributing PduCDE to human fecal microbiota [22] was detected in 34.6% (median level 5.4 log *pduC* g^−1^) of the samples followed by *B. obeum*, *V. dispar*, *C. perfringens*, and *R. gnavus* (Table 2). The occurrence of *A. hallii* and *B. obeum* was higher in the fecal microbiota of hindgut- than foregut-fermenting animals (*A. hallii*, median level 45.9 vs. 19%, *p* < 0.05; *B. obeum*, median level 50.8% vs. 4.8%, *p* < 0.001). Concurrently, the fecal microbiota of hindgut-fermenting animals harbored higher levels of *pduC* assigned to *A. hallii* (*p* < 0.01), *C. perfringens* (*p* < 0.05), and *B. obeum* (*p* < 0.001). *B. obeum* and *C. perfringens* were most often detected in feces of carnivores (Table 2) and abundance of *C. perfringens* and *B. obeum pduC* was higher in carnivores than in herbivores and omnivores (*p* < 0.001 and *p* < 0.05) (Table 2). Interestingly, *pduC* of *R. gnavus* was exclusively present in fecal microbiota of foregut-fermenting animals. While most of the tested species showed a preference for glycerol or 1,2-PD in vitro, there was no or marginal growth of *A. hallii*, *B. obeum*, *L. reuteri*, and *R. gnavus* when either substrate was provided as sole carbon source [23]. Occurrence and abundance can depend on the availability of alternative carbon sources, for example, *A. hallii* can cross-feed on the fermentation intermediates lactate and acetate [27], while *C. perfringens* is proteolytic, profiting from protein-rich substrate supply.

Together, our observations made after fecal metabolite analysis and qPCR suggest that both 1,3-PD and *pduC* can be considered as indicators that relate to *pdu*. To compare both markers, we analyzed the recurrence of 1,3-PD and *pduC* in samples collected from the same species with multiple sample points (*n* = 2–3), which were not necessarily obtained from the same animal. 1,3-PD could be repeatedly detected in 18.2% of the species while *pduC* was present more frequently (60.0%, *p* < 0.001), suggesting *pduC* as a more consistent marker of *pdu* than 1,3-PD (*p* < 0.001). Indeed, 38.5% of the samples harbored *pduC* and 1,3-PD and 46.2% only *pduC*, while only 1,3-PD was recovered from 5.8% of the samples. Neither *pduC* nor 1,3-PD was detected in 9.6% of the samples. The higher occurrence of *pduC* than of 1,3-PD might relate to the diet-dependent formation of 1,3-PD and also points out that the potential of a microbial community to confer a specific function is not necessarily indicative that the corresponding pathway is active.

### The potential for PduCDE activity is shared by few taxa

To investigate the occurrence and diversity of *pdu* harboring microbes using an untargeted approach, we generated metagenomic sequences of 25 fecal samples representing different diet types (17 herbivores, three carnivores, five omnivores) and gut physiology (11 foregut and 14 hindgut fermenters). Quality controlled reads from shotgun sequencing were assembled into scaffolds, which were subsequently used for metagenomic assembled genome (MAG) construction.

**Fig. 3:**
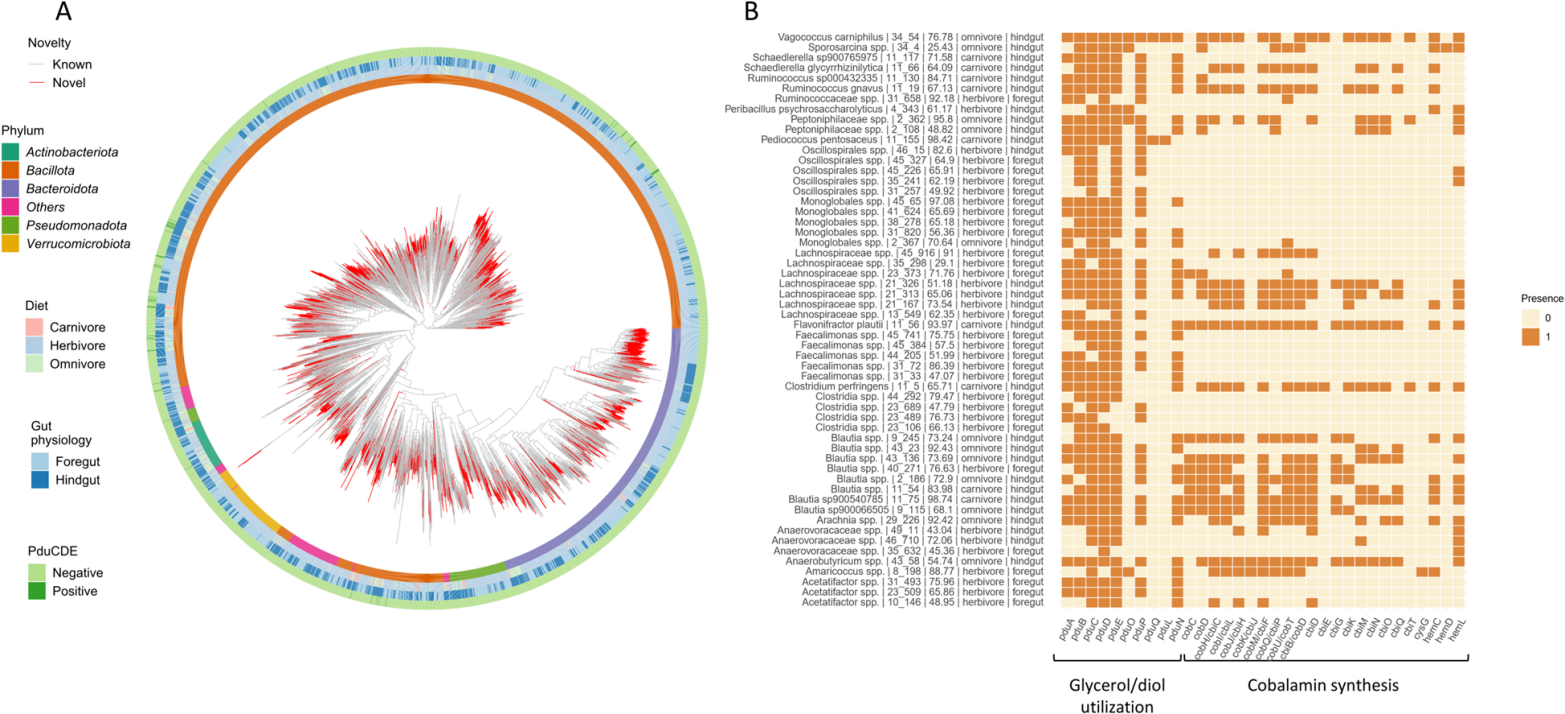
Occurrence of *pdu/cob-cbi-hem* related genes in rMAGs that harbored *pduCDE*. **A** Phylogenetic analysis of species-level representative MAGs (rMAGs). Genome sequences of rMAGs were used in phylogenetic analysis conducted by Phylophlan 3.0. The heatmaps were added via gheatmap. Shown are annotated phyla of rMAGs, presence of *pduCDE* in rMAGs, and host diet and gut physiology. Phyla that consisted of less than 100 MAGs were labeled as “Others” in the heatmap. The maximum ANI values were used for novelty categorization as described [5]. Pdu coding genes that are present in MAGs were annotated as a layer of heatmap on the phylogenetic tree. MAGs harboring at least one of *pduCDE* were labeled with “Positive”; otherwise, they were labeled with “Negative.” **B** Presence of individual genes at *pdu/cob-cbi-hem* operons in *pdu* harboring rMAGs. Genome sequences of rMAGs with > one of *pduCDE* were extracted, and the presence or absence of individual genes at *pdu/cob-cbi-hem* loci is shown. The annotation on the left side is taxon|sample number_rMAG id|completeness of rMAG|diet of host|gut physiology of host

In total, 5040 MAGs were constructed, and 4958 MAGs were annotated as belonging to 25 phyla of the domain *Bacteria* (Fig. 3A). MAGs of *Bacteria* were grouped into 4150 species-level clusters and from every cluster, the representative MAG (rMAG) with the highest Qscore was selected for phylogenetic analysis. Among all rMAGs, 61.4 and 20.0% were assigned to *Bacillota* and *Bacteroidota*, respectively (Fig. 3A). The rMAGs assigned to *Verrucomicrobiota*, *Pseudomonadota*, and *Actinomycetota* contributed 5.1, 4.5, and 3.2%, respectively. A total of 1848 rMAGs (44.5%) were suggested as novel species. These rMAGs belonged to 18 phyla, which were mainly *Bacillota* (*n* = 1094), *Bacteroidota* (*n* = 362), and *Verrucomicrobiota* (*n* = 114) similar to a previous study that discovered > 50% of novel species from the fecal microbiota of wild and captive *Mammalia*, *Aves*, *Reptilia*, and *Osteichthyes*, suggesting that wild animals living in captivity are a promising source of novel microbes [5].

Genes retrieved from MAGs and assemblies were clustered, quantified, and annotated to construct a gene catalog from captive wild animal fecal microbiota. To obtain taxa with the potential to metabolize glycerol and 1,2-PD, we identified rMAGs that harbored at least one gene of *pduCDE* and extracted *pdu* and *cob-cbi-hem* related genes (Table S3) of those rMAGs from the gene catalog. In total, 56 rMAGs (1.3% of all rMAGs) possessed at least one subunit encoding gene of *pduCDE* (Fig. 3B). The recovery in approx. 1% of rMAGs suggested *pduCDE* as a microbial function shared by few taxa in agreement with results of qPCR and as reported in humans [22, 23]. The 56 rMAGs were derived from 21/25 samples (84%), which was similar to the *pduC* occurrence identified with qPCR (85%). The majority (*n* = 54) of rMAGs harboring *pduC*, *pduD*, and/or *pduE* were assigned to *Bacillota*, one to *Actinomycetota* (*Propionibacteriaceae* family) and one to *Pseudomonadota* (*Rhodobacteraceae* family). The predominant family harboring *pduC* was *Lachnospiraceae* including the genera *Blautia*, *Ruminococcus*, *Anaerobutyricum*, *Acetatifactor*, and *Faecalimonas.* Twelve of 56 rMAGs were annotated to species level, including *R. gnavus* and *C. perfringens* (Fig. 3B), validating the results obtained by qPCR*.* No rMAG from the species *L. reuteri*, a frequently occurring contributor of *pduC*, was reconstructed due to its low abundance as shown by qPCR.

### Pdu function generally co-occurs with cobalamin synthesis and the potential for BMC formation

In the extensively researched *L. reuteri*, most of the strains that possessed *pdu* also harbored *cob-cbi-hem* [6] and only few genomes with only *pdu* and lacking *cob-cbi-hem* were detected [26]. A similar co-occurrence has been reported for other *Lactobacillaceae*, including *Loigolactobacillus coryniformis* [28] and *Furfurilactobacillus rossiae* [29], and several pathogens [30] while gene synteny was not as preserved for *A. hallii* [31].

We tested for the co-occurrence of *pdu* and *cob-cbi-hem* operons (genes are listed in Table S3) in the 56 rMAGs that harbored *pduCDE* (Fig. 3B) and found that 19 rMAGs possessed ≥ 10 genes encoding cobalamin production enzymes (*cob-cbi-hem*) including *C. perfringens*, *R. gnavus*, and *F. plautii.* Besides producing the co-factor for PduCDE, cobalamin synthesis is considered as an example of the “Black Queen Hypothesis” (BQH), which suggests a selective advantage to microorganisms that lose energetically costly functions. Only a minority population of “helper microbes” may maintain the ability to provide cobalamin as an indispensable public good to the community [32]. In agreement, only 179 of 4150 rMAGs had the potential for cobalamin synthesis based on the presence of 10 or more genes on *cob-cbi-hem* loci. These 179 rMAGs included the 19 rMAGs that also harbored *pdu* suggesting that taxa, which possessed *pdu* and *cob-cbi-hem*, contribute an important community function.

PduCDE driven substrate transformation occurs in a BMC [10]. Using *pduA* and *pduB* as marker genes for the potential to initiate BMC formation, the majority of rMAGs (83.9%) that were positive for *pduCDE* harbored genes encoding PduA or PduB, highlighting the co-occurrence of PduCDE and BMC formation in species present in fecal microbiota of captive wild animals (Fig. 3B). From a biotechnological perspective, BMC offer solutions for metabolic and biomedical engineering that can increase enzyme stability and catalysis rates [33, 34]; captive animal microbiota can be considered as a novel source of BMC.

### The potential for glycerol/1,2-PD transformation and final metabolite production was higher in carnivore than herbivore fecal microbiota

**Fig. 4:**
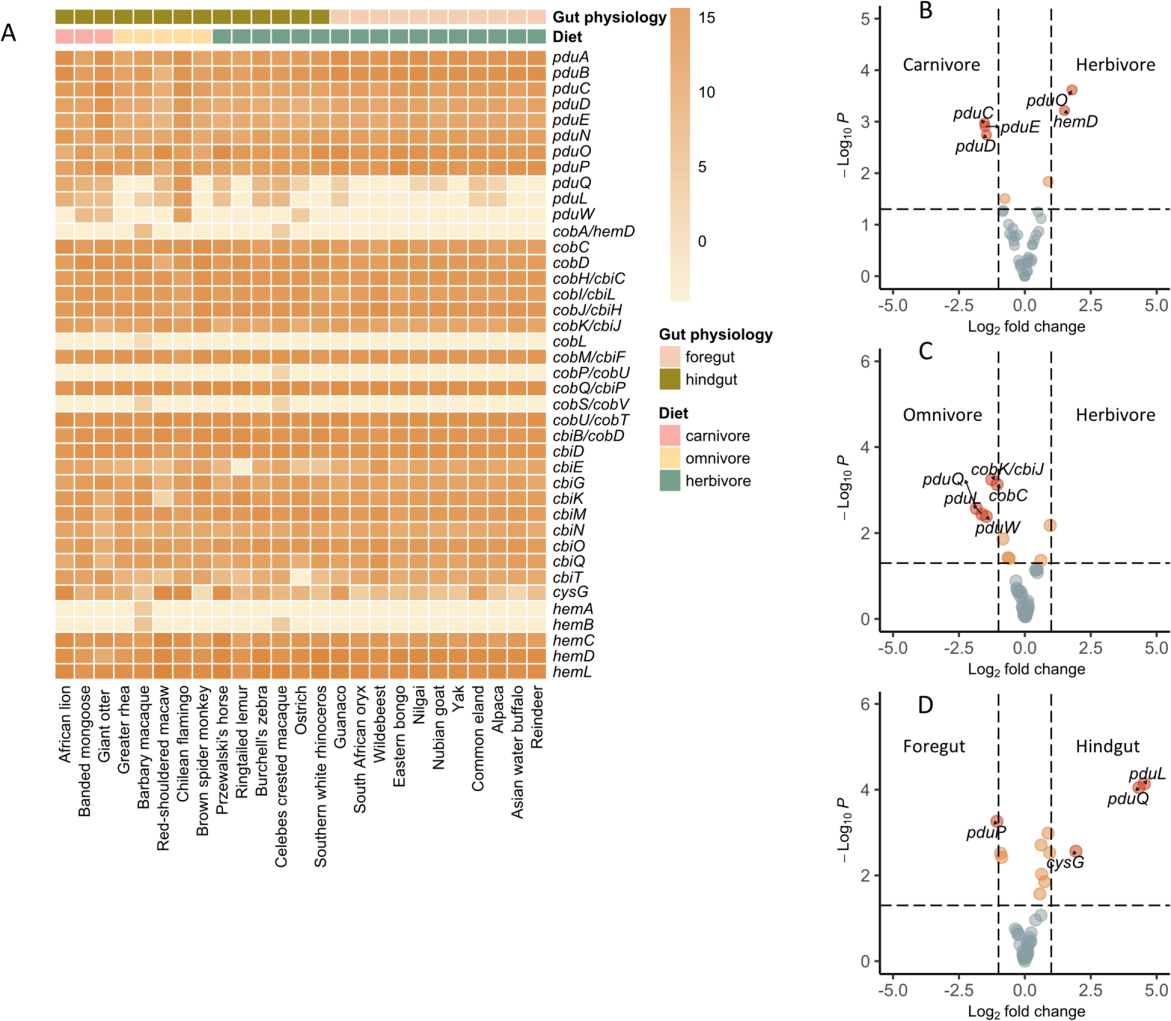
Abundance of genes of the *pdu/cob-cbi-hem* operons in the gene catalog of fecal microbiota of captive wild animals. **A** Count matrix of genes in *pdu/cob-cbi-hem* operons. Counts of individual genes of the *pdu/cob-cbi-hem* operons were transformed by variance-stabilizing transformations through Deseq2. **B**/**C**/**D** Differential analysis of gene abundance of *pdu/cob-cbi-hem* operons in fecal microbiota of animals with different diet or gut physiology. The abundance of 42 genes from the *pdu/cob-cbi-hem* operons was extracted from the gene catalog and was compared: herbivore vs. carnivore (**B**), herbivore vs. omnivore (**C**), and hindgut vs. foregut (**D**)

To gain understanding on how diet and gut physiology impact abundance of genes related to the *pdu* (including genes homologous to *pduABN*) and *cob-cbi-hem* operons in metagenomes of fecal microbiota, we extracted relevant genes (Table S3) from the gene catalog and constructed host-dependent *pdu/cob-cbi-hem* profile. Based on functional profiles, *pduCDE* and most other genes of *pdu* were present in samples of all 25 animal species as suggested by qPCR analysis indicating the common potential of captive animal microbiota for PduCDE activity (Fig. 4A). In addition, the majority of *cbi*, *cob*, and *hem* genes of the anaerobic (except *cbiA*), aerobic (except *cob*), C_3_, and C_4_ pathways of cobalamin biosynthesis, respectively, were detected in fecal microbiota of the 25 animal species (Fig. 4A). Using DESeq2 [35], we tested for differences in abundance of genes of *pdu/cob-cbi-hem* profiles of fecal microbial communities. The abundance of *pduCDE* was significantly higher in carnivore than herbivore microbiota, indicating a higher potential for PduCDE activities (Fig. 4B). Higher abundance of *pduLQ* in omnivore than herbivore fecal microbiota (Fig. 4C) and in microbiota of hindgut compared to foregut fermenters (Fig. 4D) suggested a higher potential for the formation of final metabolites of the Pdu pathway (e.g., propanol and propionate, or 1,3-PD and hydroxypropionate, Fig. 1). Except for *hemD*, two *cob* genes, and *cysG*, most of genes related to cobalamin biosynthesis did not differ in abundance in fecal microbiota of hosts with different diet and gut physiology (Fig. 4B, C, D).

### *pdu*/*cob-cbi-hem* related genes were more diverse in carnivore than herbivore fecal microbiota

To investigate whether composition and diversity of the microbial community related to the presence of *pdu*, we generated taxonomic profiles from metagenomic sequences with mOTUs [36] and normalized, analyzed, and visualized phylum abundance with Phyloseq [37]. In parallel, we extracted gene abundance related to the *pdu* (including genes homologous to *pduABN*) and *cob-cbi-hem* operons (Table S3) from the gene catalog to construct a *pdu/cob-cbi-hem* functional profile. We calculated and compared diversity indices from mOTUs and a *pdu/cob-cbi-hem* functional profile with the R package “vegan.” Due to the small number of samples, the diversity indices of PDU functional profile were combined for comparison of carnivores/omnivores to herbivores.

**Fig. 5:**
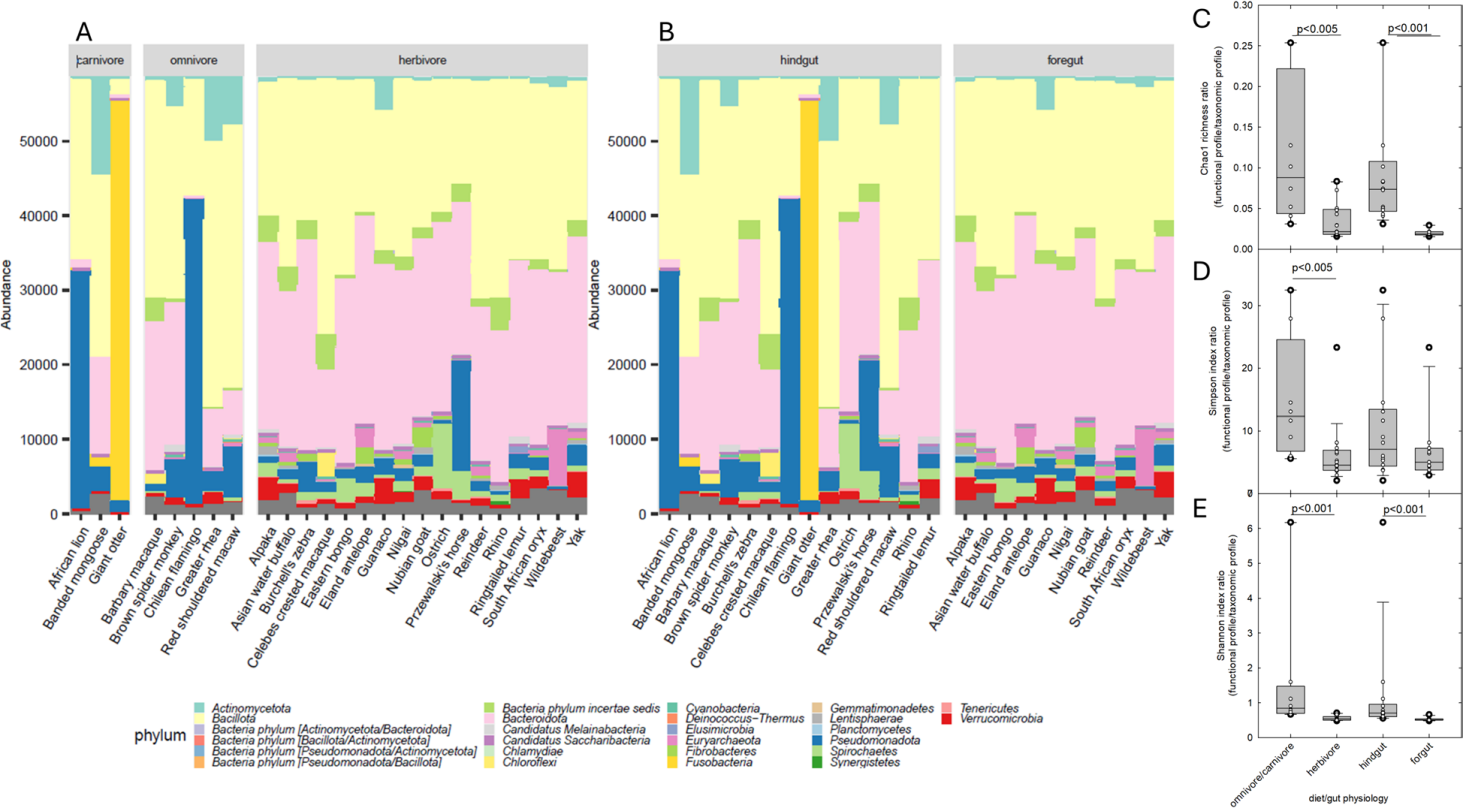
Fecal microbiota composition and diversity. Taxonomic profiles of fecal microbiota from 25 animal species were constructed with mOTUs. Samples were grouped by diet (**A**) and fermentation organs (**B**). *Candidatus Melainabacteria* and *Candidatus Saccharibacteria* are provisional names for characterized but uncultured organisms. The ratio of richness (**C**), evenness (**D**), and alpha diversity (**E**) of *pdu/cob-cbi-hem* functional profile to mOTU taxonomic profile were calculated

In total, 7798 mOTUs were identified and assigned to 26 phyla, comparable to 27 phyla of rMAGs. In accordance with rMAGs phylogeny, the fecal microbiota of foregut- and hindgut-fermenting herbivores and omnivores was dominated by *Bacteroidota* and *Bacillota* in most samples (Fig. 5A, B). Alpha- and β-diversity of fecal microbiota were analyzed based on mOTU abundance. The α-diversity of fecal microbiota of herbivores was more rich, even, and diverse than those of carnivores/omnivores, based on Chao1, Simpson’s evenness, and Shannon indices, respectively (non-parametric Wilcoxon test, *p* < 0.05, Fig. S1). Both Chao1 and Shannon indices were higher in fecal microbiota of foregut than hindgut fermenters (*p* < 0.01, Fig. S1) and β-diversity was significantly influenced by diet and gut physiology (permutational multivariate analysis of variance based on Bray–Curtis index of the relative abundances of mOTUs, *p* < 0.05, Fig. S2) in agreement with other studies [2, 5, 24]. Lower compositional diversity of fecal microbiota of hindgut-fermenting carnivores compared to herbivores may be due to a faster gut passage/lower retention time of the dietary material [1] reducing the possibility for effective microbial degradation and fermentation.

While α-diversity of *pdu/cob-cbi-hem* functional profiles of fecal microbiota was not different across diets and gut physiology (Fig. S3), β-diversity based on Bray–Curtis index was significantly related to diet and gut physiology (*p* < 0.01, Fig. S4). In previous studies, α-diversity of human fecal microbiota (= hindgut-fermenting omnivores) was positively correlated with the diversity of selected glycosyl hydrolase, and compositional diversity related strongly to functional diversity in thermal spring sediments [38, 39]. For fecal microbiota of wild animals living in captivity, compositional α-diversity was not reflected in *pdu/cob-cbi-hem* diversity with a significantly higher ratio of *pdu/cob-cbi-hem* to compositional diversity of omnivores/carnivores to herbivores, or hindgut to foregut fermenters (Fig. 5C, D, E). These observations suggest the community diversity is not predictive of the richness of *pdu/cbi-cob-hem*, which point at high relevance of functions encoded by *pdu/cbi-cob-hem* especially in hindgut-fermenting omnivores and carnivores.

### PDU driven glycerol and 1,2-PD metabolism in vitro is predicted by genetic and metabolic biomarkers

As our observational data suggested that the majority of fecal microbial communities was capable of PduCDE activity, albeit with higher potential if collected from hindgut-fermenting carnivores, we compared the impact of 1,2-PD and glycerol addition on substrate metabolism and overall fermentation activity during in vitro fermentation. Fecal samples (*n* = 23) from nine hindgut-fermenting carnivores, omnivores, and herbivores (each *n* = 3) and three foregut-fermenting herbivores that were included in metagenomic sequencing were selected for in vitro fermentation (Table S1). Samples were anaerobically incubated for 24 h using Macfarlane as control medium (MF-CON), which contains complex plant polysaccharides, protein and peptide sources and mucin (Suppl. Methods). Macfarlane medium was supplemented with glycerol (MF-GLYC) and 1,2-PD (MF-12PD, both 60–70 mM), which were compared to MF-CON. We collected samples at 0, 6, and 24 h during the fermentation for analysis of substrate utilization and metabolite formation.

Microbiota of hindgut fermenters produced higher level of total SCFA in MF-CON than foregut-fermenting microbiota (*p* < 0.05, Table S4), while microbiota of carnivores produced higher levels of total SCFAs than herbivore (*p* < 0.05) and/or omnivore (*p* < 0.05) microbiota at all conditions. In agreement with previous studies, supplementation with 1,2-PD increased the proportion of propionate and decreased the proportion of butyrate produced by carnivore, herbivore, and hindgut-fermenter microbiota (*p* < 0.05, Table S4), while glycerol supplementation reduced the proportion of propionate (*p* < 0.05, Table S4) [22, 40].

**Fig. 6:**
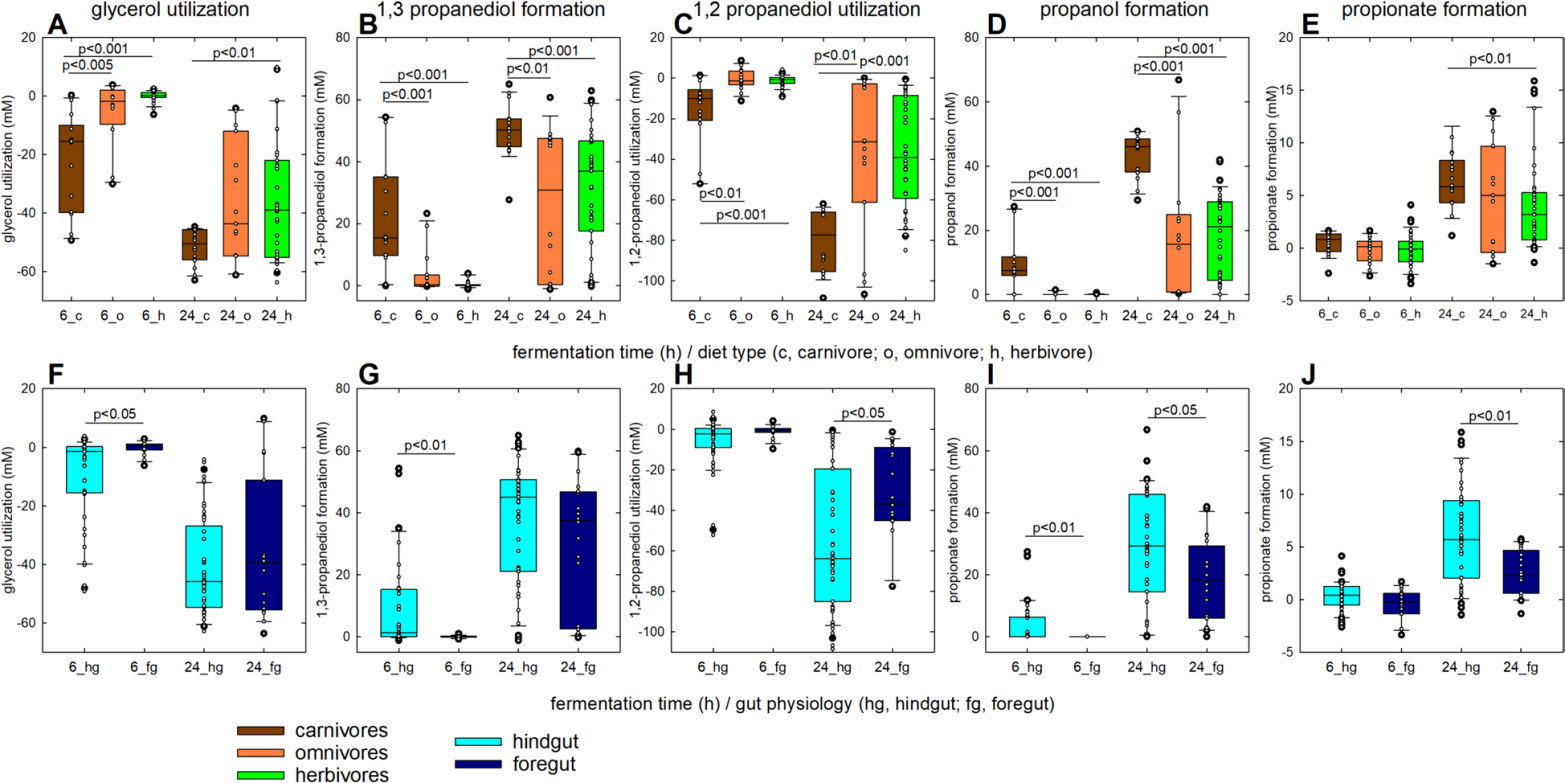
Metabolite production from glycerol and 1,2-PD from fermented fecal samples. Substrate utilization and metabolite formation in samples collected from carnivores, omnivores and herbivores (**A**-**E**) and hindgut and foregut fermenters (F-J) were determined with HPLC-RI. The consumption of glycerol (**A**/**F**) and 1,2-PD (**C**/**H**) and during fermentation with fecal microbiota of animals following different diets is shown, as well as the corresponding metabolites, 1,3-PD (**B**/**G**), propanol (**D**/**I**), and propionate (**E**/**J**). The fermentations were performed with Macfarlane medium (MF-CON) or Macfarlane medium with glycerol (**A**, **B**, **F**, **H**) or 1,2-PD (**C**-**E**, **H**-**J**) anaerobically at 37 °C for 24 h. Two samples from each species (exception of the Chilean flamingo) were used, and each sample was fermented in triplicates. The figures were plotted using SigmaPlot, box plots show median, 25 and 75% percentiles, and dots indicate individual values

With MF-GLYC and MF-12PD, the microbiota of carnivores consumed ~90% of the provided glycerol and 1,2-PD (Fig. 6A, C), respectively, which was consistent with higher abundance of *pduC/pduCDE* based on qPCR and DeSeq2 analysis. Carnivore fecal microbiota consumed more glycerol or 1,2-PD at 6 and/or 24 h (*p* < 0.01) and produced more 1,3-PD and propanol (Fig. 6A, B, C, D) than omnivores and herbivores. Hindgut-fermenting microbiota had higher glycerol/1,2-PD consumption and 1,3-PD/propanol formation than foregut fermenters at 6 or 24 h (*p* < 0.01) (Fig. 6F, G, H, I). With MF-12PD, carnivores and hindgut fermenters produced more propionate with 1,2-PD than herbivores or foregut fermenters (*p* < 0.01) (Fig. 6E, J), corresponding to higher abundance of *pduLQW* as suggested by DeSeq2 analysis.

**Fig. 7:**
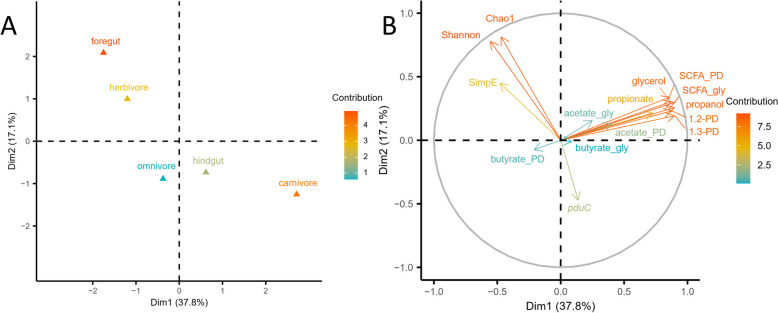
Factor analysis of mixed data including quantitative and qualitative variables. Factor analysis of mixed data (FAMD) was used to analyze association between all variables; both qualitative (diets and gut physiologies; **A**) and quantitative variables (α-diversity indices, substrate consumption, metabolite production, and individual SCFA concentrations; **B**) are shown

Factor analysis of mixed data (FAMD) was conducted to investigate the association between quantitative data including content of total and individual SCFAs, substrate consumption, metabolite production, absolute *pduC* abundance, and α-diversity indices, with qualitative data, including diet and gut physiology. The first two principal components explained 54.9% of the variation (Fig. 7). Combining qualitative and quantitative variables, α-diversity indices were positively related to fecal microbiota derived from herbivores and foregut fermenters, and negatively to carnivores, hindgut fermenters, and *pduC* abundance (Fig. 7). Carnivore microbiota was positively related to substrate utilization and metabolite concentrations except for butyrate production from MF-12PD. The consumption of glycerol and 1,2-PD positively related to the production of 1,3-PD, propionate, and propanol. These results re-emphasize that *pduC* abundance and Pdu related activity rather are a function of hindgut-fermenting carnivores than of foregut fermenter/herbivores.

## Conclusion

The trait-based approach used in this study established the relationship between diversity and selective functioning of the microbial community from animal fecal samples and allowed the prediction of a common function in animal fecal microbiota based on genetic and metabolic biomarkers. Our results suggest that gut physiology and animal diet influence gut microbial composition and the potential of gut microbiota to utilize glycerol/1,2-PD and to produce cobalamin. We conclude that abundance of *pduC/pduCDE* can be used as biomarkers to predict potential glycerol or 1,2-PD utilization via Pdu activity from a complex microbial community. In addition, this study identifies the gut microbiota of wild animals as a largely untapped resource for the discovery of novel microbes and functions that might be of relevance in biotechnological approaches.

## Material and methods

### Sample collection

Fresh fecal samples were collected using a noninvasive method by zookeepers in 2020 and 2021. Fecal matter was collected using a sterile tool, transferred into sterile 50-ml containers, and immediately frozen at − 20 °C until further processing. No contact with animals occurred during sample collection. In total, 104 fresh fecal samples from 55 animal species were obtained: ten samples from hindgut-fermenting carnivores, 25 samples from hindgut-fermenting omnivores, 42 samples from foregut-fermenting herbivores, and 27 samples from hindgut-fermenting herbivores (Table S1). The first batch of samples included 49 samples from Givskud Zoo and 7 from Copenhagen Zoo, both in Denmark. At the second collection, 48 fecal samples were obtained from Givskud from the same animal species as the first batch with the exception of the Chilean flamingo.

### DNA isolation from fecal samples

DNA from 0.2 to 0.3 g frozen fecal samples was isolated using the FastDNA Spin Kit for Soil (MP Biomedicals) following the instructions with exceptions. Briefly, samples were lysed twice at 6.0 m∙s^−1^ for 40 s using Lysing Matrix E tubes and a FastPrep-24 instrument (MP Biomedicals). DNA was eluted with nuclease-free water. The quality of DNA was evaluated by agarose gel electrophoresis to test DNA degradation, and the concentration of DNA was measured with Qubit 2.0 by Novogene Sequencing Europe.

### Quantitative PCR

Quantitative PCR (qPCR) was conducted to quantify selected bacterial groups harboring *pduC* that were previously shown to be prevalent in human fecal microbiota and artificial intestinal microbiota that was derived from animals, including *A. hallii*, *L. reuteri*, *B. obeum*, *R. gnavus*, *F. plautii*, and* V. dispar* [22]. In addition, primers were generated targeting *pduC* of *C. perfringens* (Table S2, Suppl. Methods).

Standards were prepared, and qPCR were run as previously described [41]. Briefly, a tenfold dilution series of each linearized plasmid containing the target gene was included in the run to determine the linear range and the limits of detection (Table S2). Each run contained negative controls without template DNA. PCR protocols were run for 40 cycles as outlined in Suppl. Methods followed by melting curve analysis. For results below the detection limit of the standards, ½ log gene counts at the detection limit were used for statistical analysis.

### Metagenomic sequencing and data processing

DNA samples from 25 captive animal species were selected based on DNA quality, 1,3-PD concentration of samples, and host properties, and sent for shotgun sequencing using an Illumina HiSeq (Novogene Sequencing Europe). These samples encompassed different gut physiology (11 from foregut and 14 from hindgut) and diets (17 from herbivores and three/five from carnivores/omnivores) (Table S1).

Metagenomic sequencing datasets were processed as previously described [42]. Briefly, BBMap (v.38.71) was used to quality control sequencing reads from all samples by removing adapters from the reads, removing reads that mapped to quality control sequences (PhiX genome), and discarding low-quality reads (*trimq* = *14*, *maq* = *20*, *maxns* = *1*, and *minlength* = *45*). Quality-controlled reads were merged using *bbmerge.sh* with a minimum overlap of 16 bases, resulting in merged, unmerged paired, and single reads. The reads from metagenomic samples were assembled into scaffolded contigs (hereafter scaffolds) using the SPAdes assembler (v3.15.2) [43] in metagenomic mode. Scaffolds with a length of ≥ 500 bp were used for gene calling using prodigal (v2.6.3, *-c -q -m -p meta*) [44].

Scaffolds were length-filtered (≥ 1000 bp) and quality-controlled reads from each metagenomic sample were mapped against the scaffolds of each sample. Mapping was performed using BWA (v0.7.17-r1188; *-a*) [45]. Alignments were filtered to be at least 45 bp in length, with an identity of ≥ 97% and a coverage of ≥ 80% of the read sequence. The resulting BAM files were processed using the *jgi_summarize_bam_contig_depths* script of MetaBAT2 (v2.12.1) to compute within- and between-sample coverages for each scaffold [46]. The scaffolds were binned by running MetaBAT2 on all samples individually (*–minContig 2000* and *–maxEdges 500*). Metagenomic bins were annotated with Anvio (v7.1.0) [47] and quality-controlled using the CheckM (v1.0.13) [48] lineage workflow (completeness ≥ 50% and contamination < 10%) to generate 4958 prokaryotic metagenomic assembled genomes (MAGs). Completeness was predicted using Prokka (v1.14.6) [49], and MAGs were taxonomically annotated with GTDBtk (v1.7) [50]. A representative set of MAGs (rMAGs, *n* = 4150) was generated by clustering all MAGs using the dRep (v3.2.2, *S_ani* = *0.95*) [51] dereplicate workflow.

The phylogenetic relationship of 4150 rMAGs was inferred with a maximum likelihood alignment-based approach with PhyloPhlAn3 [52]. Visualization and annotation of the tree was performed with GGTREE [53].

### Construction of a captive animal fecal microbial gene catalog

Genes from the complete set of MAGs (*n* = 7,985,556), genes from the scaffolds (*n* = 58,355,880), and manually selected *pduCDE* genes (*n* = 166) were clustered at 95% identity using CD-HIT (v4.8.1) with the parameters *-c 0.95 -M 0 -G 0 -aS 0.9 -g 1 -r 0 -d 0 -b 1000* while keeping the longest sequence as representative. Representative gene sequences (*n* = 46,443,565) were aligned against the KEGG database (release April 2021) using DIAMOND (v2.0.15) [54] and filtered to have a minimum query and subject coverage of 70% and requiring a bitScore of at least 50% of the maximum expected bitScore (i.e., score when the reference sequence is aligned against itself) to construct a gene catalog.

Quality-controlled metagenomic sequencing reads were aligned against the gene catalog and read abundances were normalized to a gene length of 1000 bp. Gene abundances were divided by the median of the abundances of ten universal single copy marker genes (COG0012, COG0016, COG0018, COG0172, COG0215, COG0495, COG0525, COG0533, COG0541, COG0552) to derive per-cell abundances and multiplied by 1000 [55, 56].

### Taxonomic profiling and microbiota diversity analysis

Version 3.0.1 of the mOTUs database was augmented with the 4958 prokaryotic MAGs using the *mOTUs-extender* tool (https://github.com/motu-tool/mOTUs-extender). Metagenomic sequencing samples were then taxonomically profiled using mOTUs [36] with the extended database as reference.

Phylum abundances were analyzed and plotted with Phyloseq [37] after rarefication with the median sum of all mOTUs. Species richness (Chao1), evenness (Simpson’s evenness), and diversity (Shannon’s) indices were calculated after rarefaction of mOTUs abundance with minimum sum of all mOTUs via the vegan package as measures of α-diversity [57]. Beta-diversity was determined with non-metric multidimensional scaling (NMDS) based on dissimilarity matrix of Bray–Curtis index calculated from the relative abundances of mOTUs using the vegan package [57].

### Identification of *pdu*/*cob-cbi-hem* related genes and analysis of functional profiles

For rMAGs with at least one of three *pduCDE genes*, the presence of *pdu*/*cob-cbi-hem* related genes (Table S3) were tested to explore the co-occurrence of *pdu* with *cob-cbi-hem* genes. To determine the distribution of operons among different animals, the abundance of *pdu*/*cob-cbi-hem* related genes (Table S3) in metagenomic samples was extracted from the gene catalog to construct a *pdu*/*cob-cbi-hem* functional profile.

The *pdu* genes encoding PduGH and PduABB’JKNTU to construct the shell of BMC in *Salmonella enterica* [58] have not been assigned to KO numbers and were therefore not identified in the gene catalog that was annotated based on the KEGG database. We extracted genes encoding proteins that were homologous to PduABN from our gene catalog and added them into *pdu*/*cob-cbi-hem* functional profile (Table S3). In detail, we added EutM and EutK, which were identified a homologous proteins to PduA in *Salmonella typhimurium* and to CcmK in *Synechocystis* sp. strain PCC6803 [59, 60]. EutL and EutS were homologs to PduB [60], and EutN and CcmL were homologs to PduN [60].

Gene counts the *pdu*/*cob-cbi-hem* functional profile were rarefied with the minimum sum of all genes within animal samples after rounded into integers for α-diversity indices calculation. Alpha-diversity indices were calculated with rarefied gene counts of *pdu*/*cob-cbi-hem* functional profiles. The relative abundance of rarefied gene counts was used for β-diversity ordination with NMDS based on Bray–Curtis index. Rounded gene counts of the *pdu*/*cob-cbi-hem* functional profile were used for functional differential analysis based on diets or gut physiology by Deseq2 (v1.36.0), and the results were processed by LFC shrinkage with “ashr” [61]. The functional profile processed via DESeq2 was corrected with variance stabilizing transformation to remove the dependence of the variance on the mean for count matrix plotting [35]. The significance level was 0.05, and the log2-fold change cutoff was 1.

### Anaerobic batch fermentations to determine the PDU-dependent metabolic activity of selected fecal microbiota

To assess the potential PDU activity of selected fecal microbiota, anaerobic fermentation of fecal samples was performed under standardized conditions using anaerobically prepared modified Macfarlane medium [62] containing complex peptides and carbohydrates as nutrients (Suppl. Methods) that mimicked substrate availability in intestinal environments and was suitable for the diverse samples included in this study. Macfarlane medium does not contain free glycerol or fucose [63] to disrupt fermentation. Fecal samples (*n* = 23) from 12 animal species (three hindgut-fermenting carnivores, omnivores, and herbivores and three foregut-fermenting herbivores, Table S1) were selected for anaerobic fermentation. Macfarlane media was adapted to conditions for batch fermentations by increasing buffer capacity [64] and was supplemented with approx. 60 mM glycerol and 70 mM 1,2-PD (both Merck). Fecal slurries were prepared by resuspending fecal material in anaerobic peptone water to prepare a 10% (w/v) fecal suspension in an anaerobic chamber (Baker Ruskinn). The slurry supernatant was inoculated into fresh Macfarlane media to obtain a 1% inoculation. Samples were incubated at 37 °C for 24 h and collected at 0, 6, and 24 h for substrate and metabolite analysis. Two independently collected fecal samples were used from each species except for the Chilean flamingo. Two or three independent biological replicates were run for every fecal sample.

### Substrate and metabolite analysis using high-performance liquid chromatography with a refractive index detector (HPLC-RI)

A 1260 Infinity II LC with RI (Agilent) was used to determine substrates and metabolites in fecal samples and during anaerobic fermentation of fecal samples, including glycerol, 1,2-PD, 1,3-PD, propanol, propionate, acetate, and butyrate as described previously [41]. Metabolites were extracted from 200 to 300 mg of animal feces with 5 mM H_2_SO_4_; supernatants from batch fermentations were analyzed directly using a Hi-Plex-H column (300 × 7.7 mm) that was connected to a guard column (7.7 × 50 mm, 8 µm, all Agilent). The mobile phase was 5 mM H_2_SO_4_ with a flow rate of 0.6 ml∙min^−1^ at 40 °C. External standards were used for quantification. The minimum detection limit was 0.01 mM.

### Statistical analysis

Every dataset was tested for normality using the Shapiro–Wilk test. Alpha-diversity indices were compared using nonparametric Wilcoxon tests to identify differences between diets and gut physiologies. Permutational multivariate analysis of variance was used to test whether the difference in composition of the microbial communities was explained by the grouping of these samples in different diets or gut physiologies. Carnivore and herbivore samples were combined because of the limited number of samples for metagenomic sequencing. The differences of *pduC* abundance in the fecal microbiota of animals with different diets and gut physiologies were compared by the nonparametric Wilcoxon test. The occurrence of *pduC* dependent on diet and gut physiology and the proportion of *pduC* from a given species within different diets or gut physiologies were compared with the *z* test.

The significance of the differences in substrate consumption, metabolite production, and SCFAs at 6 and 24 h was assessed with Kruskal–Wallis test followed by Mann–Whitney pairwise test for microbiota derived from carnivores, omnivores, and herbivores, and with Mann–Whitney test to compare between microbiota of hindgut and foregut fermenters. Factor analysis of mixed data (FAMD) was used to analyze association between all variables, both quantitative and qualitative variables via FactoMineR [65] and results were visualized via factoextra [66].

## Supplementary Information

Additional file 1: Supplement methods.

## Data Availability

Metagenomic datasets are available at ENA, accession number: PRJEB62607, link for reviewers: https://www.ebi.ac.uk/ena/browser/view/PRJEB62607. All other data is presented in the manuscript or in supplementary materials.
