# Supplementary material for: Trait-based study predicts glycerol/diol dehydratases as a key function of the gut microbiota of hindgut-fermenting carnivores
Source: Microbiome. 2024 Sep 19;12:178. doi: 10.1186/s40168-024-01863-4 (PMC11414229; doi:10.1186/s40168-024-01863-4)
Supplement: Supplementary file 2 — Additional file 1: Supplement methods. [file 40168_2024_1863_MOESM1_ESM.pdf]

Supplementary data

Trait-based study predicts glycerol/diol dehydratases as a key function of the gut microbiota of hindgut-fermenting carnivores

Qing Li[1*], Hans-Joachim Ruscheweyh[2], Lærke Hartmann Østergaard[1], Micael Libertella[1], Kim Skalborg Simonsen[3], Shinichi Sunagawa[2], Alberto Scoma[1], Clarissa Schwab[1#]

[1]Department of Biological and Chemical Engineering, Functional Microbe Technology Group, Aarhus University, Denmark

[2]Department of Biology, Institute of Microbiology and Swiss Institute of Bioinformatics, ETH Zürich, Vladimir-Prelog-Weg 4, 8093 Zürich, Switzerland

[3]Givskud Zoo - Zootopia, Løveparkvej 3, 7323 Givskud

*current address: National Food Institute, Technical University of Denmark, Kgs. Lyngby, Denmark

#corresponding author, email: schwab@bce.au.dk

The authors declare no competing financial interest

Keywords

Diet, gut physiology, glycerol diol dehydratase, metagenomic sequence, biomarker

Supplement methods

Preparation of anaerobic media and peptone water

Macfarlane media was adapted to conditions for batch fermentations by increasing the amount of KH_2PO_4 and $NaHCO_3$ two-fold (1) and was supplemented with 100 mM of glycerol or 1,2-PD (both from Sigma-Aldrich) (2). The medium composition (g L^{-1}) was: 1.0 cellobiose, 1.0 xylan, 1.0 arabinogalactan, 0.5 inulin, 1.0 soluble potato starch, 3.0 casein acid hydrolysate, 5.0 bacto™ tryptone, 1.5 meat extract, 4.5 yeast extract, 4.0 mucin, 0.4 bile salt, 0.05 hemin, 0.61 $MgSO_4$, 0.1 $CaCl_2{\cdot}2H_2O$, 0.2 $MnCl_2{\cdot}4H_2O$, 0.005 $FeSO_4{\cdot}7H_2O$, 0.1 $ZnSO_4{\cdot}7H_2O$, 2.0 KH_2PO_4, 6.0$NaHCO_3$, 4.5 NaCl and 4.5 KCl. One ml of Tween 80 and vitamin solution were added. Short-chain fatty acids were added to supply initial nutrients for a final concentration of 33 mM acetate, 9 mM propionate and 1 mM of isobutyrate, isovalerate and valerate (3). All components of the nutritive medium were purchased from Merck. The pH of the medium was adjusted to 6.8 with 5 M NaOH. Cysteine-HCl was added to the medium to a final concentration of 1.0 $g{\cdot}L^{-1}$ after boiling and gassing with CO_2. The medium was dispensed in 20 mL portions in 50 mL serum flasks under flowing CO_2 into 50 mL serum flasks containing magnetic stirrers and closed with butyl septum stoppers and aluminum caps before autoclaving. The following steps were carried out in the absence of oxygen using an anaerobic chamber (Baker Ruskinn, Concept 500) flushed with N_2, CO_2 and H_2.

Primer design and application for amplification of *pduC* assigned to *Clostridium perfringens*

The primers targeting *pduC* were designed based on PduC encoding nucleotide sequences associated with *B. obeum* ATCC 29174 (EDM88959.1), *A. hallii* DSM 3553 (EEG37600.1), *L. reuteri* DSM 20016 (ABQ83986.1), *Flavonifractor plautii* JCM 32125 (QIA30165.1), *R. gnavus* CAG:126 (CCZ66961.1), and *V. dispar* ATCC 17748 (EEP66242.1)(2).

The primers targeting *pduC* of *Cl. perfringens* were designed based on the overlapped region of *pduC* sequences from six species/strains available in ad (*Clostridium estertheticum subsp. estertheticum* strain DSM 8809, *Clostridium cochlearium* NCTC13027, *Clostridium chauvoei* 12S0467, *Clostridium gasigenes* strain CGAS001, *Clostridium baratii* strain CDC51267, *Clostridium perfringens* strain CPI 18-6), with an expected product size of 330 bp.

Primer specificity was tested using PCR and using DNA isolated from human feces (available from previous studies), wild captive animal feces and *Clostridium* species including *Cl. perfiringens* FMT 1001/1006, *Clostridium difficile* DSM 27147, *Clostridium paraputrificum* DSM 2630, *Clostridium ramosum* DSM 1402, *Clostridium butyricum* FMT 1007, *Clostridium clostridioforme* DSM 933, *Clostridium sporogenes* FMT 528, *Cl. baratii* FMT 558, and *Paraclostridium bifermentans* FMT 577. The desirable product was obtained with DNA of *Cl. perfringens* as the template. The nucleotide sequence of the amplicon was verified by Sanger sequencing with identity of 97% to 99%.

qPCR protocols

Reactions were set up by preparing a master mix by using 5 µL of iTaq Universal SYBR Green Supermix (2X) (Bio-Rad, California, USA), 1 µL of forward and reverse primers, and 2 µL of nuclease free water, and adding 1 µL of template DNA for a final volume of 10 µL per reaction. Every reaction was run in duplicates. The qPCR analysis was run with CFX Connect Real-Time PCR Detection System (Bio-Rad CFX9 system). All target genes except *pduC* of *Clostridium perfringens* were amplified in a 2-step program: 95.0°C for 3.0 min initial denaturation, followed by 40 cycles of 95.0°C for 10 sec and 60.0°C for 30 sec.

For *pduC* of *C. perfringens*, the amplification cycle protocol was a 3-step program with the following cycle conditions: 95.0°C for 3.0 min initial denaturation, followed by 40 cycles of 95.0°C for 10 sec and 55.0°C for 20 sec and 60°C for 30 sec.

Table S1. Summary of sample collection, fecal metabolites and abundance of *pduC* and analysis overview. Samples were obtained from Givskud Zoo and Copenhagen Zoo. Fecal metabolites were analysed with HPLC-RI, abundance of *pduC* was determined with qPCR.

**Host**					**Fecal metabolites (mM)**				**Abundance of *pduC* from selected taxa (cells·g^{-1})**							**Analysis information**	
sample nr.	common name	species	diet	gut physiol-ogy	aceta te	propio nate	butyr ate	1.3-PD	*A. hallii*	*L. reute ri*	*R. gnavus*	*V. dispa r*	*C.perfri ngens*	*B. obeum*	Sum	MG	Fermen tation
11	Banded mongoose	*Mungos mungo*	carnivo re	hindgut	60,6	18,8	10	4,6	6,8	4,7	ND	ND	ND	6,2	6,9	Y	Y
85	Banded mongoose	*Mungos mungo*	carnivo re	hindgut	16,1	ND	3,9	5,6	ND	ND	ND	ND	ND	ND	ND		Y
18	Boa constrictor	*Boa constrictor*	carnivo re	hindgut	31,2	ND	ND	ND	5,1	4,9	ND	5	ND	4,9	5,6		
27	Giant otter	*Pteronura brasiliensis*	carnivo re	hindgut	39,1	ND	6,7	7,7	ND	6,5	ND	ND	8,7	4,9	8,7	Y	Y
72	Giant otter	*Pteronura brasiliensis*	carnivo re	hindgut	31,3	ND	4,7	6,3	ND	4,8	ND	ND	9,8	ND	9,8		
65	Humboldt penguin	*Spheniscus humboldti*	carnivo re	hindgut	68,7	ND	ND	ND	ND	4,8	ND	4,9	9,5	4,9	9,5		
6	African lion	*Panthera leo*	carnivo re	hindgut	13,5	ND	6,7	5	ND	4,8	ND	ND	9,1	ND	9,1	Y	Y
103	African lion	*Panthera leo*	carnivo re	hindgut	66,2	41	22,8	ND	ND	4,8	ND	ND	9,1	4,9	9,1		Y
82	Grey wolf	*Canis lupus*	carnivo re	hindgut	5,6	ND	ND	6,2	ND	ND	ND	ND	ND	4,9	4,9		
30	Grey wolf	*Canis lupus*	carnivo re	hindgut	8,6	ND	ND	ND	ND	6,3	ND	ND	8,3	5	8,4		
76	Alpaca	*Lama pacos*	herbivo re	foregut	23,2	ND	3,1	ND	5,1	ND	ND	ND	ND	ND	5,1		
41	Alpaca	*Lama pacos*	herbivo re	foregut	23,3	ND	4	ND	ND	ND	ND	ND	ND	ND	ND	Y	
26	American bison	*Bison bison*	herbivo re	foregut	22,5	0,2	3,4	ND	ND	6,2	ND	ND	ND	ND	6,2		
81	American bison	*Bison bison*	herbivo re	foregut	14,8	ND	3	5,1	ND	4,7	ND	ND	ND	ND	4,7		
37	Bactrian camel	*Camelus bactrianus*	herbivo re	foregut	31,4	0,7	5,8	ND	ND	4,8	ND	ND	ND	ND	4,8		

78	Bactrian camel	*Camelus bactrianus*	herbivore	foregut	37,5	ND	4,3	6,5	ND	4,9	ND	ND	ND	4,9	5,2		
5	Blackbuck	*Antilope cervicapra*	herbivore	foregut	23	ND	3,4	ND	ND	ND	ND	4,9	ND	ND	4,9		
84	Blackbuck	*Antilope cervicapra*	herbivore	foregut	19,9	ND	3,5	5,2	ND	ND	ND	ND	ND	ND	ND		
62	African buffalo	*Syncerus caffer*	herbivore	foregut	25,4	ND	4	ND	ND	ND	ND	4,9	ND	ND	4,9		
15	African buffalo	*Syncerus caffer*	herbivore	foregut	36,4	0,5	4,9	ND	ND	ND	ND	ND	ND	ND	ND		
44	Asian water buffalo	*Bubalus bubalis*	herbivore	foregut	13,7	ND	3,6	ND	ND	4,9	ND	ND	ND	ND	4,9	Y	
64	Asian water buffalo	*Bubalus bubalis*	herbivore	foregut	12,3	ND	3,7	ND	ND	4,8	4,8	ND	ND	ND	5,1		
80	Common eland	*Taurotragus oryx*	herbivore	foregut	4,9	ND	ND	6	ND	4,8	ND	ND	ND	ND	4,8		
40	Common eland	*Taurotragus oryx*	herbivore	foregut	47,9	4,5	3,9	ND	ND	4,9	ND	5	ND	ND	5,2	Y	
70	Dwarf zebu	*Bos taurus indicus*	herbivore	foregut	10,4	ND	2,9	4,9	ND	ND	ND	ND	ND	ND	ND		
88	Eastern bongo	*Tragelaphus eurycerus*	herbivore	foregut	58,2	9,5	5,7	ND	ND	6,5	ND	ND	ND	ND	6,5		Y
23	Eastern bongo	*Tragelaphus eurycerus*	herbivore	foregut	39,7	5,6	4,8	4,6	ND	ND	6	ND	ND	ND	6	Y	Y
79	Giraffe	*Giraffa camelopardalis rothschildi*	herbivore	foregut	23,1	ND	3,8	6	ND	ND	ND	ND	ND	ND	ND		
56	Giraffe	*Giraffa camelopardalis rothschildi*	herbivore	foregut	41,4	4,1	6,3	ND	ND	ND	ND	ND	ND	ND	ND		
20	Giraffe	*Giraffa camelopardalis rothschildi*	herbivore	foregut	46,4	4,9	4,9	ND	ND	ND	ND	ND	ND	ND	ND		

8	Guanaco	Lama guanicoe	herbivore	foregut	26,6	ND	4	ND	5,1	ND	4,8	ND	ND	ND	5,3	Y	Y
102	Guanaco	Lama guanicoe	herbivore	foregut	44,3	1,7	6,7	ND	ND	ND	ND	ND	ND	ND	ND		Y
55	Hippo	*Hippopotamus amphibius*	herbivore	foregut	8,5	ND	ND	ND	ND	ND	ND	5	ND	ND	5		
14	Pygmy hippo	*Choeropsis liberiensis*	herbivore	foregut	13,4	ND	3,1	5	5	4,8	ND	ND	ND	4,9	5,4		
89	Pygmy hippo	*Choeropsis liberiensis*	herbivore	foregut	10,1	ND	3,1	5,8	ND	6,4	ND	ND	ND	ND	6,4		
31	Nilgai	*Boselaphus tragocamelus*	herbivore	foregut	28	ND	4,2	ND	ND	ND	4,7	ND	ND	ND	4,7	Y	
67	Nilgai	*Boselaphus tragocamelus*	herbivore	foregut	5,2	ND	ND	6,1	ND	4,9	ND	ND	ND	ND	4,9		
35	Nubian goat	*Capra hircus*	herbivore	foregut	26,4	ND.5	4,1	ND	ND	4,8	ND	ND	ND	ND	4,8	Y	
53	Okapi	*Okapia johnstoni*	herbivore	foregut	62,5	5	8,8	ND	5,1	4,9	ND	ND	ND	ND	5,3		
25	Ouessant sheep	*Ovis aries*	herbivore	foregut	15,4	ND	3,2	ND	5	4,8	ND	ND	ND	ND	5,2		
77	Ouessant sheep	*Ovis aries*	herbivore	foregut	35,4	0,8	3,7	5,6	ND	6,4	ND	ND	ND	ND	6,4		
45	Reindeer	*Rangifer tarandus*	herbivore	foregut	18,1	ND	2,9	ND	5,1	ND	ND	ND	ND	ND	5,1	Y	
12	Sable antelope	*Hippotragus niger*	herbivore	foregut	34,1	ND.9	3,8	ND	ND	ND	ND	ND	ND	ND	ND		
58	Sable antelope	*Hippotragus niger*	herbivore	foregut	27,2	ND	4,8	9	ND	4,9	ND	5	ND	ND	5,2		
10	South African oryx	*Oryx gazella*	herbivore	foregut	22,5	ND	3,5	ND	ND	4,8	ND	ND	ND	ND	4,8	Y	
60	South African oryx	*Oryx gazella*	herbivore	foregut	25,3	0,5	5,3	ND	ND	ND	ND	5	ND	ND	5		

22	Watussi cow	*Bos taurus*	herbivore	foregut	31,6	0,4	5,2	ND	ND	ND	ND	ND	ND	ND	ND		
100	Watussi cow	*Bos taurus*	herbivore	foregut	19,9	ND	3,5	ND	ND	4,9	ND	ND	ND	ND	4,9		
13	Wildebeest	*Connochaetes gnou*	herbivore	foregut	32,6	1,5	4,1	ND	7	4,9	ND	ND	ND	ND	7	Y	Y
59	Wildebeest	*Connochaetes gnou*	herbivore	foregut	34	0,6	4,3	ND	ND	4,8	ND	ND	ND	ND	4,8		Y
38	Yak	*Bos grunniens*	herbivore	foregut	17,3	ND	3,9	ND	7,1	6,4	ND	ND	ND	ND	7,1	Y	
68	Yak	*Bos grunniens*	herbivore	foregut	28,5	0,2	4,7	5,6	ND	4,8	4,8	ND	ND	ND	5,1		
47	Bronze turkey	*Meleagris gallopavo*	herbivore	hindgut	13,1	ND	ND	ND	5,1	4,8	ND	ND	ND	ND	5,3		
98	Bronze turkey	*Meleagris gallopavo*	herbivore	hindgut	81,6	ND	3,3	5,7	ND	4,9	ND	ND	ND	ND	4,9		
1	Capybara	*Hydrochoerus hydrochaeris*	herbivore	hindgut	55,3	10,6	4,3	ND	7,1	6,5	ND	ND	ND	ND	7,2		
106	Capybara	*Hydrochoerus hydrochaeris*	herbivore	hindgut	47,2	3,5	2,9	ND	5,1	4,9	ND	5	ND	ND	5,5		
57	Capybara	*Hydrochoerus hydrochaeris*	herbivore	hindgut	37,1	3,9	ND	ND	ND	4,8	ND	ND	ND	4,9	5,2		
50	Donkey	*Equus asinus*	herbivore	hindgut	24,8	4,6	5	ND	5	4,8	ND	ND	ND	ND	5,3		
94	Asian elephant	*Elephas maximus*	herbivore	hindgut	21,2	0,8	4,3	ND	ND	ND	ND	ND	ND	ND	ND		
7	Asian elephant	*Elephas maximus*	herbivore	hindgut	24,3	1,7	5,1	ND	6,8	4,8	ND	ND	ND	4,9	6,8		
17	Gorilla	*Gorilla gorilla*	herbivore	hindgut	46,1	5,1	7,1	ND	8	4,9	ND	ND	ND	6,8	8,1		
96	Gorilla	*Gorilla gorilla*	herbivore	hindgut	36,9	3,1	7,7	ND	8,9	ND	ND	ND	ND	7,3	8,9		

39	Black lemur	*Eulemur macaco*	herbivore	hindgut	19,7	ND	3,8	9,9	8,4	4,8	ND	ND	ND	4,9	8,4		
86	Black lemur	*Eulemur macaco*	herbivore	hindgut	27	ND	4,3	5,9	9,4	4,8	ND	4,9	ND	ND	9,4		
21	Ringtailed lemur	*Lemur catta*	herbivore	hindgut	38,1	2	7,6	ND	9,4	4,8	ND	ND	ND	ND	9,4	Y	Y
83	Ringtailed lemur	*Lemur catta*	herbivore	hindgut	29,8	1,5	5,8	5,1	ND	4,8	ND	4,8	ND	ND	5,1		Y
48	Celebes crested macaque	*Macaca nigra*	herbivore	hindgut	17,2	ND	5,3	ND	8,7	7,8	ND	ND	ND	7,3	8,8	Y	Y
66	Celebes crested macaque	*Macaca nigra*	herbivore	hindgut	22,3	1,5	6,4	ND	ND	8,3	ND	5	8,5	6,8	8,7		Y
49	Ostrich	*Struthio camelus*	herbivore	hindgut	109,4	3,9	5,3	ND	ND	4,8	ND	ND	8,8	4,9	8,8	Y	Y
101	Ostrich	*Struthio camelus*	herbivore	hindgut	56,1	11,7	5,3	ND	5,1	ND	ND	4,9	ND	4,9	5,4		Y
4	Przewalski's horse	*Equus ferus przewalskii*	herbivore	hindgut	5,1	ND	3,5	6,2	6,7	ND	ND	ND	ND	4,8	6,7	Y	
97	Przewalski's horse	*Equus ferus przewalskii*	herbivore	hindgut	26	ND	5	6,8	5,1	ND	ND	ND	ND	5	5,3		
24	Red panda	*Ailurus fulgens*	herbivore	hindgut	48,3	ND	5,8	14,6	4,9	4,7	ND	ND	ND	4,8	5,3		
73	Red panda	*Ailurus fulgens*	herbivore	hindgut	18,2	ND	6,4	6,9	5	4,8	ND	ND	ND	4,9	5,4		
54	Southern white rhinoceros	*Ceratotherium simum simum*	herbivore	hindgut	26,3	0,2	4,7	7	ND	4,8	ND	ND	ND	ND	4,8	Y	
16	Southern white rhinoceros	*Ceratotherium simum simum*	herbivore	hindgut	35,2	7,2	5,1	ND	5	4,8	ND	ND	ND	4,9	5,4		
61	Southern white rhinoceros	*Ceratotherium simum simum*	herbivore	hindgut	13,6	ND	3,8	6	ND	4,8	ND	ND	ND	ND	4,8		
99	Burchell's zebra	*Equus quagga burchellii*	herbivore	hindgut	11,9	ND	3,8	ND	ND	ND	ND	ND	ND	ND	ND		

46	Burchell's zebra	*Equus quagga burchellii*	herbivore	hindgut	22,4	ND	4,3	ND	6,6	4,9	ND	ND	ND	ND	6,6	Y	
43	Brown spider monkey	*Ateles hybridus*	omnivore	hindgut	72,4	18,1	10,5	ND	ND	4,8	ND	ND	ND	ND	4,8	Y	Y
91	Brown spider monkey	*Ateles hybridus*	omnivore	hindgut	64,2	11,9	10	5,3	ND	4,9	ND	ND	ND	ND	4,9		Y
92	Brown spider monkey	*Ateles hybridus*	omnivore	hindgut	57,8	17	9,9	ND	ND	6,1	ND	4,9	ND	4,9	6,1		
34	Chilean flamingo	*Phoenicopterus chilensis*	omnivore	hindgut	83,5	ND	5,4	5,8	ND	4,8	ND	4,9	ND	ND	5,2	Y	Y
33	Brown-nosed coati	*Nasua nasua*	omnivore	hindgut	9,6	ND	ND	7,9	ND	4,8	ND	ND	ND	4,9	5,2		
93	Brown-nosed coati	*Nasua nasua*	omnivore	hindgut	122	ND	19,8	ND	ND	ND	ND	ND	10,5	ND	10,5		
87	Brown-nosed coati	*Nasua nasua*	omnivore	hindgut	47	ND	11,7	6	ND	ND	ND	ND	ND	ND	ND		
69	Common emu	*Dromaius novaehollandiae*	omnivore	hindgut	44,1	ND	5,6	ND	ND	ND	ND	4,9	ND	ND	4,9		
36	Common emu	*Dromaius novaehollandiae*	omnivore	hindgut	44,8	ND	6,1	9,5	ND	4,9	ND	ND	ND	5	5,2		
32	Grey parrot	*Psittacus erithacus*	omnivore	hindgut	168	ND	ND	ND	ND	6,9	ND	ND	ND	4,9	6,9		
63	Grey parrot	*Psittacus erithacus*	omnivore	hindgut	153,5	ND	ND	ND	ND	4,9	ND	5	ND	ND	5,2		
9	Barbary macaque	*Macaca sylvanus*	omnivore	hindgut	37,6	6,4	8,6	ND	NA	NA	NA	NA	NA	NA	ND	Y	Y
90	Barbary macaque	*Macaca sylvanus*	omnivore	hindgut	55,7	5,5	10,7	6,3	9,3	7,8	ND	4,9	ND	4,9	9,4		Y
75	Blue-throated macaw	*Ara glaucogularis*	omnivore	hindgut	188,8	ND	ND	10,7	6,6	ND	ND	ND	ND	5	6,6		

42	Blue-throated macaw	*Ara glaucogularis*	omnivore	hindgut	42,1	ND	ND	ND	6,6	6,1	ND	ND	ND	4,9	6,7		
71	Military macaw	*Ara militaris*	omnivore	hindgut	18,9	ND	ND	4,6	ND	6,2	ND	ND	ND	4,9	6,2		Y
19	Military macaw	*Ara militaris*	omnivore	hindgut	74,2	ND	ND	5,9	6,6	4,8	ND	ND	ND	ND	6,6		
29	Red-shouldered macaw	*Diopsittaca nobilis*	omnivore	hindgut	107,3	ND	ND	6,1	6,5	4,7	ND	ND	ND	ND	6,5	Y	
105	Red-shouldered macaw	*Diopsittaca nobilis*	omnivore	hindgut	121	ND	ND	4,9	5	4,8	ND	4,9	ND	4,9	5,5		
51	Panda yellow	*Ailuropoda melanoleuca*	omnivore	hindgut	11,4	2	ND	8	4,9	4,7	ND	ND	ND	4,8	5,3		
52	Panda green	*Ailuropoda melanoleuca*	omnivore	hindgut	19,3	ND	3,7	7,1	ND	ND	ND	ND	ND	ND	ND		
104	Greater rhea	*Rhea americana*	omnivore	hindgut	44,6	0,5	5,2	4,9	ND	ND	ND	4,9	ND	ND	4,9		
2	Greater rhea	*Rhea americana*	omnivore	hindgut	19,8	ND	ND	4,5	ND	6,3	ND	ND	ND	ND	6,3	Y	
3	Spectacled bear	*Tremarctos ornatus*	omnivore	hindgut	23	ND	ND	9	ND	4,8	ND	ND	ND	ND	4,8		
95	Spectacled bear	*Tremarctos ornatus*	omnivore	hindgut	76,9	ND	9,3	7,7	5,1	ND	ND	ND	ND	ND	5,1		

MG, metagenome sequencing, ND, not detected, NA DNA isolate from sample nr. 9 was missing and not used, *F. plautii* was not amplified in the fecal samples

Table S2. **Primers used to quantify *pduC* of selected taxa using qPCR.**

Target	Primer sequences	Product size (bp)	Linear detection range (gene copies)	Reference
Anaerobutyricum hallii	F: CGTTATGCTCCATTTAATGCT<br>R:CCAAGGAGTATCATCACCATC	192	log 2.84 − log 8.84	(2)
Limosilactobacillus reuteri	F: CGTTATGCACCATTCAATGCT<br>R:CCATGGAGTATCATCACCATC	192	log 2.42 − log 10.42	(2)
Ruminococcus gnavus	F: CTGAAGGTCCGCTTTACATC<br>R: CAAACATATTGTCATAGTTCG	370	log 2.36 − log 9.36	(2)
Veillonella dispar	F: CGTTATGCACCACTTAATGCG<br>R: CCAAGGTGTATCATCACCATC	192	log 2.59 − log 9.59	(2)
Blautia obeum	F: CTGAAGGTACGTTTTACCTC<br>R: CGAACATATTGTCGTAGTTTG	370	log 2.58 − log 9.58	(2)
Clostridium perfringens	F: GTR GTT GAA ATG ATG ATG<br>R: AWG GWG TAT CRT CKC CAT	355	log 1.49 − log 8.49	this study
Flavonifractor plautii	F: CTGAAGATGCGCTTTACCTC<br>R: CGAACTACGACAACATGTTCG	370	-	(2)

Table S3. Genes located on *pdu*/*cob-cbi-hem* operons and the encoded enzymes

KO	COG	EC	Genes	Description
K19697	COG0282	2.7.2.15	*pduW*	propionate kinase
K13921	COG1454		*pduQ*	1-propanol dehydrogenase
K13922	COG1012	1.2.1.87	*pduP*	propionaldehyde dehydrogenase
K00798	COG2096	2.5.1.17	*pduO*	cob(I)alamin adenosyltransferase
K13923	COG4869	2.3.1.222	*pduL*	phosphate propanoyltransferase
K13920,K06122	COG4910	4.2.1.30,4.2.1.28	*pduE / dhbE*	glycerol / propanediol dehydratase small subunit
K06121,K13919		4.2.1.30,4.2.1.28	*pduD / dhbC*	glycerol / propanediol dehydratase medium subunit
K01699,K06120	COG4909	4.2.1.28,4.2.1.30	*pduC / dhaB*	glycerol / propanediol dehydratase large subunit
K01845	COG0001	5.4.3.8	*hemL*	glutamate-1-semialdehyde 2,1-aminomutase
K01719	COG1587	4.2.1.75	*hemD*	uroporphyrinogen-III synthase
K01749	COG0181	2.5.1.61	*hemC*	hydroxymethylbilane synthase
K01698	COG0113	4.2.1.24	*hemB*	porphobilinogen synthase
K02492	COG0373	1.2.1.70	*hemA*	glutamyl-tRNA reductase
K04031	COG4810		*eutS*	ethanolamine utilization protein EutS
K04028	COG4576		*eutN*	ethanolamine utilization protein EutN
K04027	COG4577		*eutM*	ethanolamine utilization protein EutM
K04024	COG4820		*eutJ*	ethanolamine utilization protein EutJ
K02302	COG0007,COG1648	2.1.1.107,1.3.1.76,4.99.1.4	*cysG*	uroporphyrin-III C-methyltransferase / precorrin-2 dehydrogenase / sirohydrochlorin ferrochelatase
K00768	COG2038	2.4.2.21	*cobU, cobT*	nicotinate-nucleotide--dimethylbenzimidazole phosphoribosyltransferase
K02233	COG0368	2.7.8.26	*cobS, cobV*	adenosylcobinamide-GDP ribazoletransferase
K02232	COG1492	6.3.5.10	*cobQ, cbiP*	adenosylcobyric acid synthase
K02231	COG2087	2.7.1.156,2.7.7.62	*cobP, cobU*	adenosylcobinamide kinase / adenosylcobinamide-phosphate guanylyltransferase
K05936	COG2875	2.1.1.133,2.1.1.271	*cobM, cbiF*	precorrin-4/cobalt-precorrin-4 C11-methyltransferase
K00595	COG2241,COG2242	2.1.1.132	*cobL*	precorrin-6Y C5,15-methyltransferase (decarboxylating)
K05895	COG2099	1.3.1.54,1.3.1.106	*cobK-cbiJ*	precorrin-6A/cobalt-precorrin-6A reductase
K05934	COG1010	2.1.1.131	*cobJ, cbiH*	precorrin-3B C17-methyltransferase
K03394	COG2243	2.1.1.130,2.1.1.151	*cobI-cbiL*	precorrin-2/cobalt-factor-2 C20-methyltransferase
K06042	COG2082	5.4.99.61,5.4.99.60	*cobH-cbiC*	precorrin-8X/cobalt-precorrin-8 methylmutase
K04720	COG0079	4.1.1.81	*cobD*	threonine-phosphate decarboxylase
K02226	COG0406	3.1.3.73	*cobC*	alpha-ribazole phosphatase
K02224	COG1797	6.3.5.9,6.3.5.11	*cobB-cbiA*	cobyrinic acid a,c-diamide synthase
K13542	COG0007,COG1587	2.1.1.107,4.2.1.75	*cobA-hemD*	uroporphyrinogen III methyltransferase / synthase
K16927			*cbrT*	energy-coupling factor transport system substrate-specific component

K02191	COG2242	2.1.1.196	*cbiT*	cobalt-precorrin-6B (C15)-methyltransferase
K02008	COG0619		*cbiQ*	cobalt/nickel transport system permease protein
K02006	COG1122		*cbiO*	cobalt/nickel transport system ATP-binding protein
K02009	COG1930		*cbiN*	cobalt/nickel transport protein
K02007	COG0310		*cbiM*	cobalt/nickel transport system permease protein
K02190	COG4822	4.99.1.3	*cbiK*	sirohydrochlorin cobaltochelatase
K02189	COG2073	3.7.1.12	*cbiG*	cobalt-precorrin 5A hydrolase
K03399	COG2241	2.1.1.289	*cbiE*	cobalt-precorrin-7 (C5)-methyltransferase
K02188	COG1903	2.1.1.195	*cbiD*	cobalt-precorrin-5B (C1)-methyltransferase
K02227	COG1270	6.3.1.10	*cbiB, cobD*	adenosylcobinamide-phosphate synthase

Table S4. SCFA and 1,3-propanediol levels and proportions after *in vitro* fermentation for 24 h at 37 ºC in MacFarlane medium. Fecal microbiota was fermented using MacFarlane medium (MF) that was additionally supplemented with glycerol (MF-GLYC) or 1,2-PD (MF-12PD). Levels of major SCFA acetate, propionate and butyrate were determined with HPLC-RI.

			SCFA (median; 25, and 75% quartile)						
	**Factor**	**Fermentation**	**Acetate**		**propionate**		**Butyrate**		**Total SCFA**
			Levels (µmol g^{-1})	Proportion (%)	Levels (µmol g^{-1})	Proportion (%)	Levels (µmol g^{-1})	Proportion (%)	Levels (µmol g^{-1})
**Gut physiology**	Foregut	MF	17.0;10.6;22.3	81.8;70.7;86.4	0.6;0.0;2.8	3.2;0.0;11.1	2.6;0.8;5.6	13.7;6.9;22.8	21.7;15.8;27.5
		MF-GLYC	30.2;18.2;36.1	85.8;83.7;93.5	0.0;0.0;1.0	0.0;0.0;2.2	2.5;1.9;5.0	10.3;6.0;16.2	33.8;19.8;42.1
		MF-12PD	22.9;21.0;26.2	86.9*;84.5;89.1	1.9;1.5;3.3	8.3;5.8;11.2	1.1;1.0;1.8	4.7;3.6;6.7	26.4;24.0;29.4
	Hindgut	MF	23.8*;19.5;35.6	78.1;70.1;82.9	2.8^{B};0.2;3.4	6.6^{B};0.4;12.7	4.2;2.5;7.5	14.3^{AB};10.1;19.4	33.1;23.2;48.5
		MF-GLYC	31.3;21.4;38.5	82.5;71.2;91.4	0.0C;0.0;1.4	0.0^{C};0.0;2.9	5.9;2.6;12.3	15.3^{A};8.6;25.2	40.2;22.9;49.5
		MF-12PD	22.3;19.0;32.2	77.3;70.1;83.6	5.8^{A*};3.1;10.0	16.7^{A*};11.9;25.1	1.5;0.8;2.5	5.3^{C};2.6;7.2	33.5;22.8;40.5
**Diet**	Carnivores	MF	34.7^{AB};21.6;40.3	70.1^{c};67.7;73.9	3.4^{B};0.3;5.2	7.8^{B};0.6;12.6	9.4^{ABa};6.2;15.9	22.5^{Aa};17.9;27.2	48.5^{a};32.3;55.5
		MF-GLYC	38.2^{Aa};34.1;45.1	78.8;72.6;83.1	1.3^{BC};0.0;2.7	2.9^{C};0.0;5.4	9.9^{A};5.4;16.1	20.3^{B};12.4;23.6	48.6^{a};42.6;62.6
		MF-12PD	33.3^{Ba};24.9;34.8	79.3;75.5;83.5	7.4^{A};4.4;8.3	17.5^{A};12.3;22.4	0.9^{C};0.0;2.8	2.6^{C};0.0;6.5	41.0^{a};31.4;46.0
	Omnivores	MF	22.9;19.1;34.3	80.3^{ab};75.7;89.6	3.0;1.1;4.4	10.6;2.2;13.9	2.3^{bc};0.9;6.8	8.0^{bc};4.2;14.9	28.2^{ab};21.6;45.1

	MF-GLYC	21.7^{abc};10.5;36.4	84.4;39.2;93.5	0.0;0.0;1.4	0.0;0.0;2.9	6.4;1.5;10.3	11.9;6.5;59.8	23.7^{abc};18.0;44.3
	MF-12PD	22.0^{b};17.7;24.4	76.7;66.3;88.5	5.4;0.7;10.3	18.8;3.7;28.0	1.5;1.3;2.3	6.9;4.5;7.8	28.7^{ab};20.0;36.9
Herbivores	MF	18.6;13.2;24.1	81.9^{a};75.8;86.7	1.0^{B};0.0;2.6	4.7^{B};0.0;8.7	2.9^{b};2.5;4.2	13.7^{Ab};9.5;16.0	23.1^{b};18.0;30.9
	MF-GLYC	26.0^{b};14.8;34.0	85.8;80.0;93.8	0.0^{BC};0.0;0.0	0.0^{C};0.0;0.0	3.1;1.6;4.6	11.5^{AB};5.9;20.0	30.5^{b};15.4;40.4
	MF-12PD	20.6^{bc};19.1;25.6	84.6;77.5;87.7	2.7^{A};1.7;5.5	11.5^{A};6.8;16.2	1.3;0.9;2.3	5.5^{C};4.1;7.0	26.4^{b};23.2;34.2

Amount and percentage of SCFAs from MF, MF-GLYC, and MF-12PD with fecal microbiota of animals with the same diet or gut physiology do not share a common uppercase superscript differ significantly ($p<0.05$). Amount and proportion of SCFAs from the same fermentation with fecal microbiota of carnivores, omnivores, and herbivores do not share a common lowercase superscript differ significantly ($p<0.05$). * and **represent higher amount and proportion of SCFAs with $p<0.05$ and $p<0.01$, respectively, from the same fermentation between hindgut-fermenting animals and foregut-fermenting animals. Median, 25, and 75% quartiles were calculated based on average of three independent fermentations from 23 fecal samples.

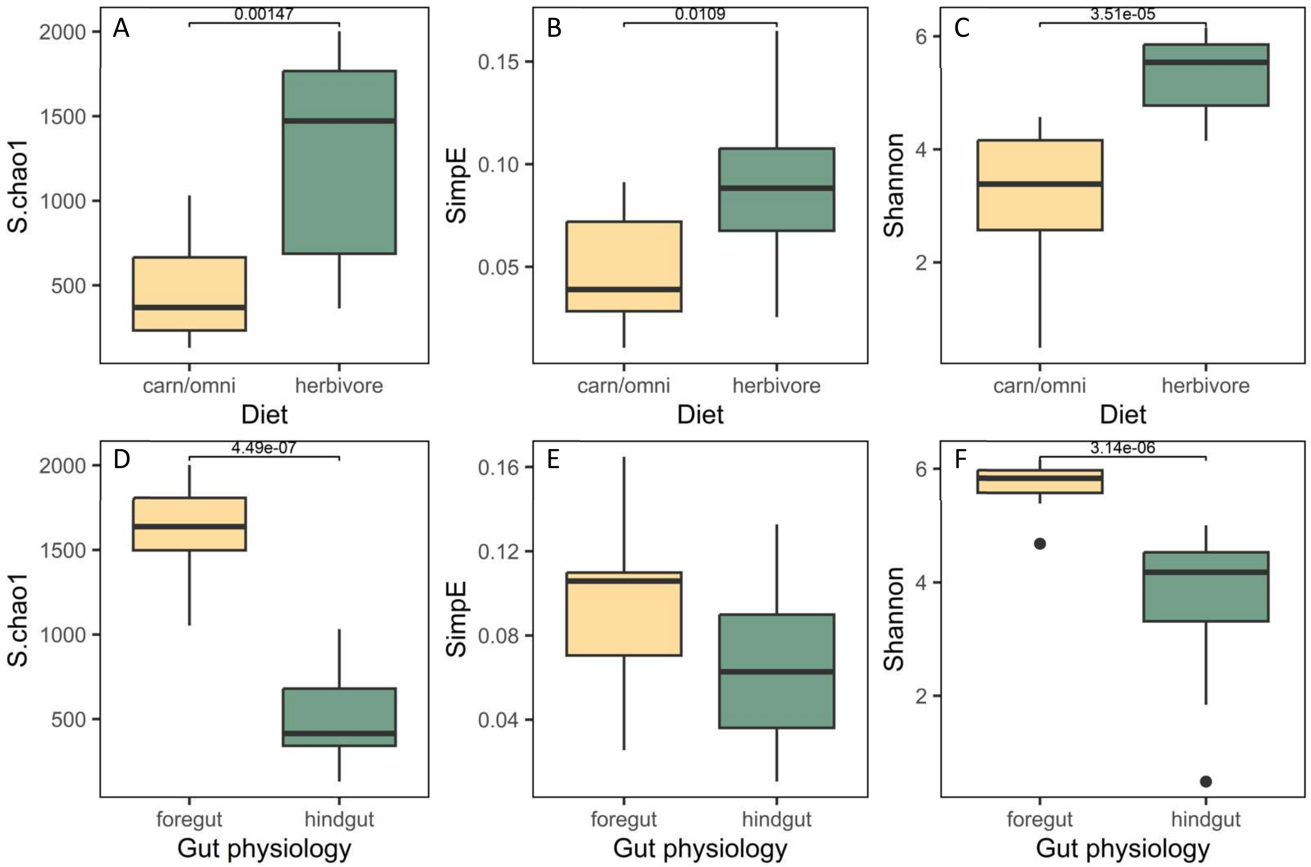

Figure S1. Richness (A/D), evenness (B/E), and alpha diversity indices (C/F) of mOTUs between diet (A/B/C) and gut physiology (D/E/F). Significant differences of every index were determined by Wilcoxon test and significant *p* values were labeled at the top of two boxes ($p<0.05$). Carnivores and omnivores were combined into one group because they had four samples for each group which was not enough to make a box plot.

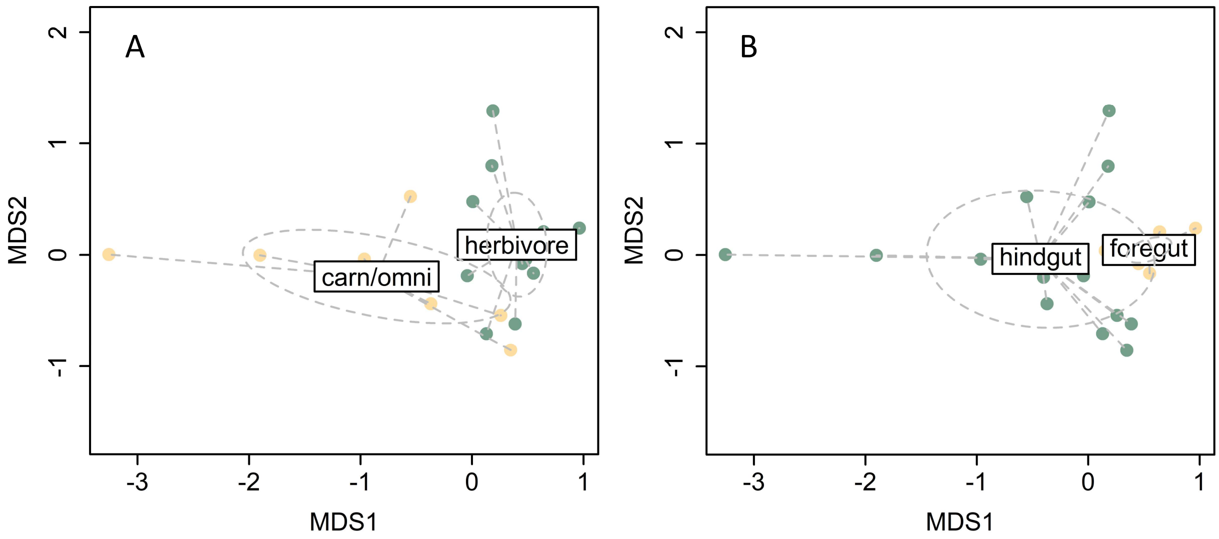

Figure S2. Beta-diversity analysis of microbial communities. Ordination of gene counts was performed via non-metric multidimensional scaling based on Bray-Curtis index calculated of the relative abundances of mOTUs. Samples were clustered by diet (A) and gut physiology (B).

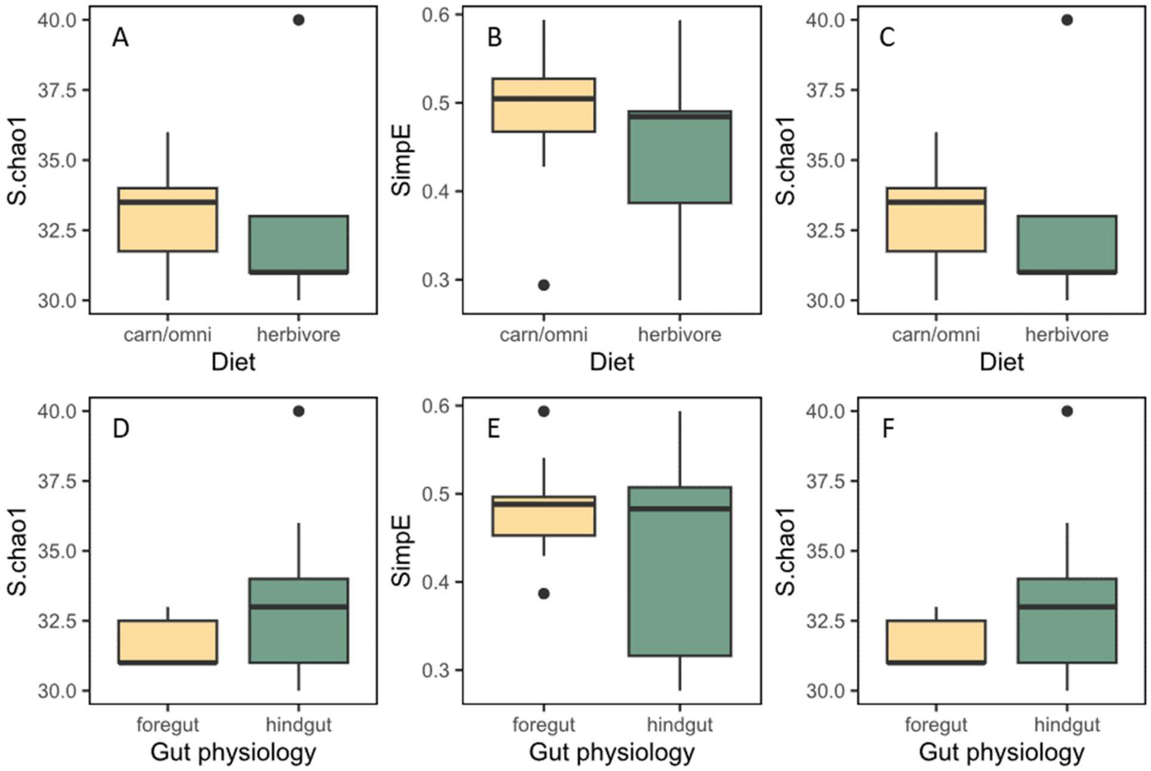

Figure S3. Richness (A/D), evenness (B/E), and alpha diversity (C/F) of the *pdu/cob-cbi-hem* functional profile between diets and gut physiologies. Carnivores and omnivores were combined into one group because they had four samples for each group which was not enough to make a box plot.

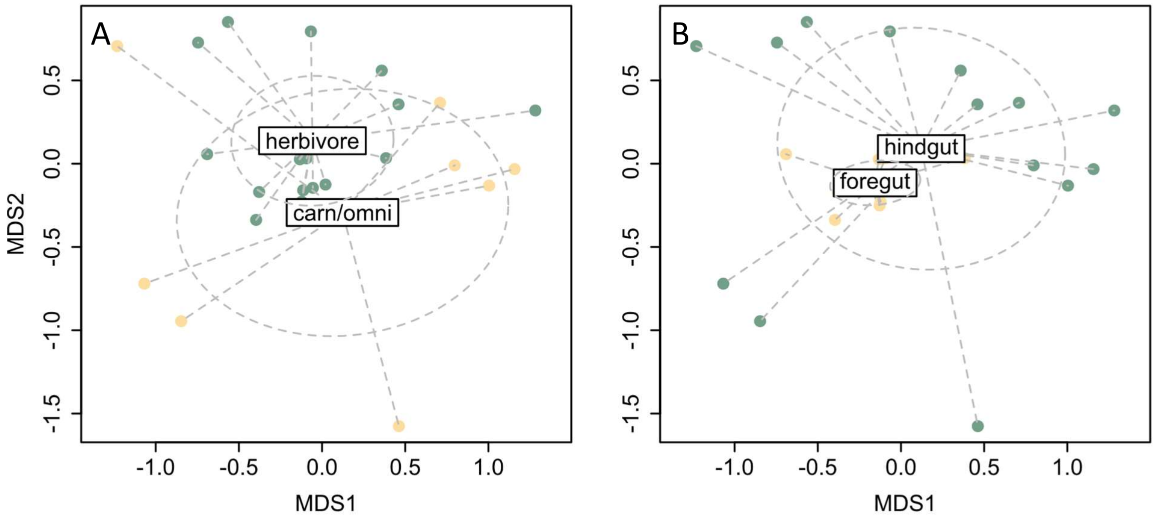

Figure S4. Ordination of function profile of *pdu/cob-cbi-hem*. Ordination of gene counts was performed via non-metric multidimensional scaling based on Bray-Curtis index calculated from the relative abundances of rarefied gene counts of *pdu/cob-cbi-hem* function profile. *Pdu/cob-cbi-hem* functional profile was clustered by diet (A) and gut physiology (B).

References

1. Bircher L, Schwab C, Geirnaert A, Lacroix C. Cryopreservation of artificial gut microbiota produced with *in vitro* fermentation technology. Microb Biotechnol. 2018;11(1):163–75.

2. Ramirez Garcia A, Zhang J, Greppi A, Constancias F, Wortmann E, Wandres M, et al. Impact of manipulation of glycerol/diol dehydratase activity on intestinal microbiota ecology and metabolism. Environ Microbiol. 2021;23(3):1765–79.

3. Duncan SH, Barcenilla A, Stewart CS, Pryde SE, Flint HJ. Acetate utilization and butyryl coenzyme A (CoA): Acetate-CoA transferase in butyrate-producing bacteria from the human large intestine. Appl Environ Microbiol. 2002;68(10):5186–90.
